# Antimicrobial Resistance: The Answers

**DOI:** 10.3389/bjbs.2026.15559

**Published:** 2026-02-06

**Authors:** Beverley C. Millar, Mary J. Cates, Marco S. Torrisi, Amanda J. Round, Aisling Warde, Colm J. Lowery, John E. Moore

**Affiliations:** 1 Northern Ireland Public Health Laboratory, Belfast City Hospital, Belfast, Northern Ireland, United Kingdom; 2 School of Biomedical Sciences, Ulster University, Coleraine, Northern Ireland, United Kingdom

**Keywords:** antimicrobial resistance (AMR), antibiotic resistance, antibiotic, innovation, new approaches, bacteriophage therapy, cystic fibrosis, policy

## Abstract

Antimicrobial resistance (AMR) has caused a global public health crisis, contributing to approximately five million deaths in 2019 and predicted deaths of approximately ten million annually by 2050. This equates to approximately 1.4-fold more deaths annually from AMR in 2050 than the entire COVID-19 pandemic to date. To tackle this AMR pandemic, regulatory and policy frameworks have been prepared at local, national and international levels with multi-faceted proposals and advances encompassing surveillance, diagnostics, infection prevention, antibiotic prescribing and variation of existing and novel treatment approaches. This narrative review primarily focuses on research and development which have been documented over the last five years in relation to therapeutic approaches at various stages in clinical development and the potential role that vaccines can play in the fight against AMR. This review provides an overview on antibacterial drugs, including novel classes of antibiotics, which have been recently approved, as well as combination antibiotic therapy and the potential of repurposed drugs. The potential role of novel antimicrobial, antibiofilm and quorum sensing inhibitors, such as antimicrobial peptides, nanomaterials and compounds from the extreme and natural environments, as well as ethnopharmacology including the antimicrobial effects of plants, spices, honey and venoms are explored. Novel therapeutic approaches are critically discussed in terms of their realistic clinical potential, detailing recent and ongoing trials to highlight the current interest of these approaches, including immunotherapy, bacteriophage therapy, antimicrobial photodynamic therapy (aPDT), antimicrobial sonodynamic therapy (aSDT), nitric oxide therapy and microbiome manipulation including faecal microbiota transplantation (FMT). The potential of predatory bacteria as living antimicrobial agents is also discussed. Importantly, there have been many technological developments which have enhanced bioprospecting and research and development of novel antimicrobials which this review draws attention to, including artificial intelligence, machine learning and Organ-on-a-Chip devices. Finally, key messages from the recent World Health Organization report into the role of vaccines against AMR provides an interesting perspective relating to prevention which can be of significance in tackling the AMR burden.

## Introduction

Antimicrobial resistance (AMR) is a global concern which continues to have an impact on public health both within healthcare and increasingly community settings, in relation to mortality and non-fatal health burden, as well as problems associated with treatability and additionally financial costs [1–3]. In 2016, the O’Neill report detailed that there were 8.2 million deaths attributed to cancer and predicted that in 2050, 10 million deaths would be attributed to AMR [1]. Due to this public health crisis, various global and national strategies have been devised and actioned to help tackle AMR using a multidisciplinary “*One Health approach*”. This framework considers the contributions to this problem attributed to human, animal and environmental factors and effective steps, which can be taken to control and limit the expansion of this problem [4].

**FIGURE 1:**
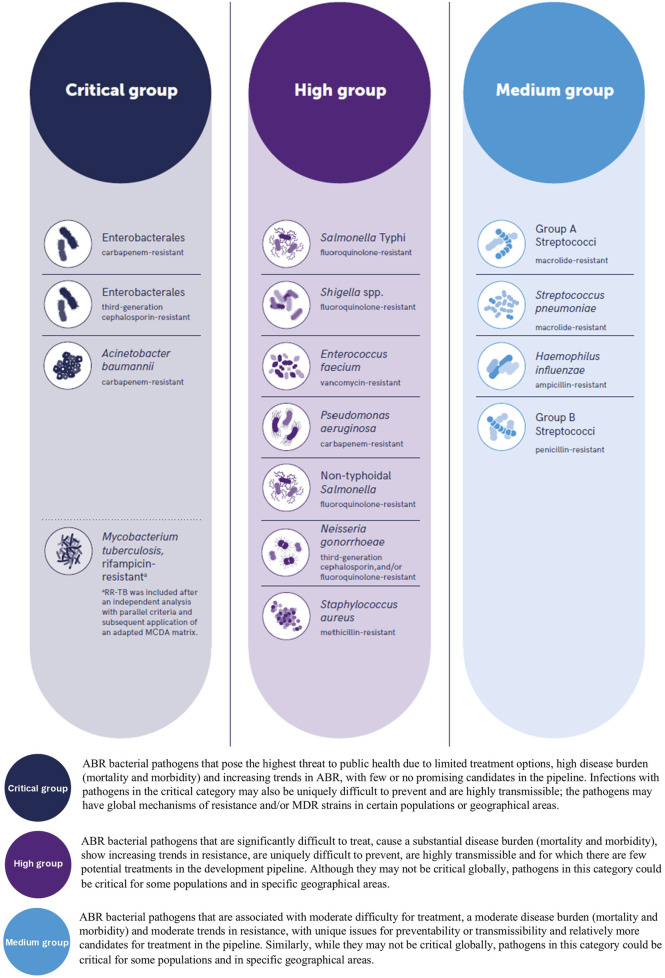
The World Health Organization (WHO) bacterial priority pathogens list, 2024. (Top) reproduced from “WHO Bacterial Priority Pathogens List, 2024” by World Health Organization (https://www.who.int/) licensed under, CC BY-NC-SA 3.0 IGO.

The consequences of AMR have impacted the clinical management of infections causing the World Health Organization (WHO) to update their “Bacterial Priority Pathogens List” (BPPL) in May 2024, seven years since their previous BPPL, during which time the pandemic of AMR has continued to lead to a global crisis particularly, but not limited to, low and middle income countries (LMIC), with some Gram-negative organisms now resistant to last-resort antibiotics [3]. The most recent BPPL lists one bacterial order, 11 named bacterial species and two Lancefield groupings of streptococci which are antimicrobial resistant and have been assigned to three priority groups, namely critical, high and medium, which are of global public health concern within vulnerable populations and LMIC, as well as organisms which are highly virulent, multidrug-resistant (MDR) and those with the ability to transfer resistance genes, including “*transmission across the One Health spectrum”*, see Figure 1 [3].

The recent BPPL highlights the severity of AMR as evidenced with the inclusion of organisms which are MDR. Drug resistant tuberculosis (DR-TB) is a primary example of an infection which continues to cause concern, particularly as treatment regimens are dependent on the causes and complex mechanisms of resistance attributed to the *Mycobacterium tuberculosis* complex [5], with various classifications of resistance being reported such as Multidrug resistant-TB (MDR-TB), Multidrug resistant or rifampicin resistant (MDR/RR-TB) and extensively drug resistant-TB (XDR-TB) which the WHO more recently clarified in terms of definitions of Pre-XDR-TB and XDR-TB to align with treatment regimens and epidemiological reporting [6]. Another pathogen which is of global concern is *Neisseria gonorrhoeae*, where resistance has been increasingly reported in relation to antibiotic empirical therapy such as ceftriaxone and more recently azithromycin [7].

Although there are fifteen families of bacterial pathogens highlighted in the recent BPPL which are deemed a priority, there are groups of individuals where there is a stark reality of AMR. One such example where AMR is of major concern, is individuals with cystic fibrosis (CF), where the repeated administration and prolonged duration of antibiotic therapy, coupled with environmental conditions in the airways, such as altered electrolyte levels, thick mucus and an acidic environment has promoted bacterial pathogens, such as *Pseudomonas aeruginosa*, to establish itself in a biofilm rather than planktonic state, further contributing to the development of AMR, particularly when such bacteria are difficult to eradicate [8]. AMR continues to have a growing significant impact in patients with cancer, where infection is common and results in the second cause of death in this patient group. The burden of AMR is of concern in these patients due to implications for them, such as increased hospital admissions and deaths, as well as associated healthcare costs [9].

The primary aims of the BPPL are multi-fold, including a focus on research and development into the development of diagnostics and novel treatments, financial input, the development of AMR policies and programmes to promote active approaches to tackling AMR, the monitoring of resistance trends, as well as embedding affordable preventative and control measures.

**FIGURE 2:**
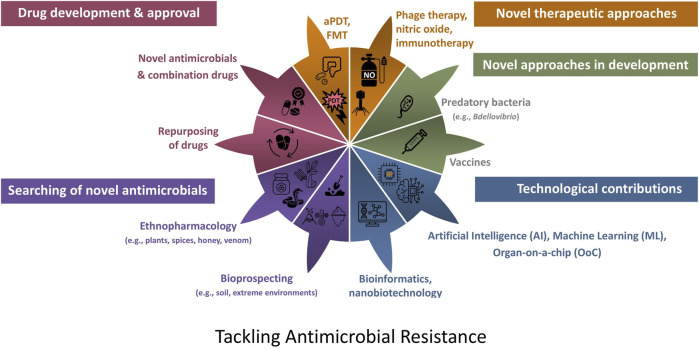
Approaches which are currently being developed to tackle the therapeutic dilemma due to antibiotic resistance. aPDT, antimicrobial photodynamic therapy; FMT, faecal microbiota transplantation.

Due to the real-world difficulties in treating infections due to bacteria with multi-faceted resistance mechanisms, the aim of this narrative review is to provide an overview of research and clinical trials which have been or continue to be conducted, primarily during the last five years in relation to searching for and developing novel therapeutic approaches to target AMR infections. The aim of this article is to provide an overview of the wealth of approaches, i.e. the answers (Figure 2) which are currently being developed to tackle the therapeutic dilemma considering the AMR global crisis and to direct readers to seminal recent articles relating to each of these approaches to further enhance their understanding and appreciation of recent research and development.

## Novel Antibacterial Drugs & Drug Repurposing

Antibiotics commonly act at one or more of the various bacterial cellular sites such as those involved in the synthesis of cell walls, protein synthesis, nucleic acid synthesis, metabolic pathways and cell membrane function, and in the case of broad-spectrum antibiotics acting at cellular sites which are common to both Gram-negative and Gram-positive bacteria [10]. Key to the research and development of novel therapeutic approaches to the treatment of antibiotic-resistant bacteria is an understanding of the various cellular mechanisms of resistance, as well as the virulence factors attributed to such organisms, as the relationship between these, including the genetic regulation of these two components is intertwined [11].

As highlighted in the O’Neill review, research and development into novel antibiotics is challenging, primarily due to associated developmental costs and predicted lack of revenue from subsequent sales. This review stated that “The total market for antibiotics is relatively large: about 40 billion USD of sales a year, but with only about 4.7 billion USD of this total from sales of patented antibiotics”. Hence, coupled with the potential for the subsequent development of AMR, without incentives, the pharmaceutical industry is reluctant to invest in this market [1].

### Recently Approved Antibiotics

Two recent seminal articles provide a comprehensive overview of the antibacterial drugs which have been approved by the United States Food and Drug Administration (US FDA) and the European Medicines Agency (EMA) during the period 2012-2022 [12, 13] and it has been reported that only twenty antibiotics, seven β-lactam/β-lactamase inhibitor combinations and four non-traditional antibacterial drugs have been launched worldwide during the last 10 years [14]. A recent evaluation of antibacterial drugs, particularly targeting those on the WHO priority list, which have been recently approved by the FDA and EMA, as well as those currently in the clinical trials pipeline, highlights that the majority of drugs are derivatives of currently available antibiotic classes and as such may succumb to similar resistance mechanisms which have been historically observed [15].

It is interesting to note two recent deemed “*First-in- class*” antibiotics approved, namely lefamulin and gepotidacin. Lefamulin (Xenleta™), is a semi-synthetic pleuromutilin and its mechanism of action is the blocking of bacterial ribosomal protein synthesis by means of interfering with the bacterial 50S RNA subunit [16]. Lefamulin was approved by the FDA (August 2019) and EMA (July 2020) followed by the Medicines and Healthcare products Regulatory Agency (MHRA), granting market authorisation in the UK in January 2021 for the treatment of community-acquired bacterial pneumonia. Until 2019, employment of pleuromutilins, was limited in human medicine to the topical treatment of impetigo and staphylococcal skin infections with retapamulin. However, market authorisation for retapamulin was withdrawn by the EMA at the request of the marketing authorisation holder, leaving the new antibiotic, lefamulin, as the sole agent within this class of antibiotic with a licence and indication in human medicine. The licensing of this pleuromutilin in human medicine creates a new dynamic, where the historical backdrop of pleuromutilins were exclusively a class of antibiotics used solely in veterinary medicine, with tiamulin and valnemulin, as licenced in the UK, for the treatment of swine dysentary caused by *Brachyspira hyodysenteriae*, complicated by the anaerobes, *Fusobacterium* and *Bacteroides*, as well as the atypicals, including *Mycoplasma* infections. Also, this class of antibiotic is typically used against *Mycoplasma* spp. and avian intestinal spirochetosis caused by *Brachyspira* in poultry [17]. The arrival of lefamulin in human medicine potentially creates a new route of transmission of pleuromutilin- resistance organisms developing in human medicine and spreading zooanthropogenically (reverse zoonosis) to livestock. As with other classes of antibiotics, cross-resistance may occur between other members of the pleuromutilin class and lefamulin [18].

Zooanthropogenic spread of bacterial pathogens has been documented, particularly with livestock and companion animals and methicillin-resistant *Staphylococcus aureus* (MRSA) [19], with the potential to compromise the antibiotic efficacy of important classes of veterinary lincosamides, phenicols, streptogramins, as well as the veterinary pleuromutilins. The equilibrium of potential pathogen transmission involving the ebb and flow effect of zoonosis and anthroponosis coupled with AMR, creates a new dynamic for further investigation under the One Health initiative. This changing topography on the licensing of antibiotics for humans requires careful epidemiological monitoring of antibiotic susceptibility in both human and veterinary medicine, coupled with robust antimicrobial stewardship to ensure longevity of effectiveness with the pleuromutilins against AMR for both our human and animal patients.

Gepotidacin, was approved by the FDA on 25 March 2025 for the treatment of female and adolescent uncomplicated urinary tract infections. This antibiotic is a first in the class of triazaacenaphthylene antibiotics whose mechanism of action is inhibition of bacterial DNA replication by inhibiting the bacterial topisomerase enzymes, namely the B subunit of DNA gyrase (topoisomerase II), as well as topoisomerase IV [20]. This novel antibiotic has a number of properties of interest such as the availability of an oral medication, potential therapeutic use in the treatment of other infections due to its broad activity against Gram-negative and Gram-positive organisms, including urogenital gonorrhoea, as observed in a recent clinical trial [21] and the fact that multiple mutations would be required in both enzymes to result in the development of resistance [20].

### FDA Legislation to Promote the Development of Novel Antibiotics

In July 2012, The FDA Safety and Innovation Act (FDASIA) became a regulatory legislation [22, 23]. One important aspect of this legislation was that the FDA could facilitate and expedite the development and review of new drugs. Title VIII within the FDASIA refers to “*Generating Antibiotic Incentives Now* (GAIN)”. The primary aim of GAIN is to offer incentives to promote the development of certain antimicrobial drugs which can result in attainable concentrations in humans to either inhibit or kill fungal and bacterial infections such as those caused by antimicrobial-resistant organisms or emerging pathogens known to cause serious or life-threatening infections [24]. A drug which qualifies for a qualified infectious disease product (QIDP), will be granted two incentive policies: an additional 5 years of market exclusivity and a priority review during the review phase.

Following approval in June 2021 by The National Medical Products Administration, China [25], the FDA recently, September 2023, granted the pharmaceutical company MicuRX, a QIDP as well as fast track designation in relation to their oxazolidinone antimicrobial drugs contezolid (oral), and contezolid acefosamil (prodrug, intravenous) for the treatment of Gram-positive infections in severe diabetic foot infection without concomitant osteomyelitis [26]. Research is active in the potential use of contezolid in the treatment of several infections including tuberculosis due to its efficacy and safety profile [27], the treatment of methicillin-sensitive *S*. *aureus* infective endocarditis with cerebrovascular complications [28] and lung abscess due to *S. aureus* [29]. Interestingly, contezolid has been successful in the treatment of antibiotic-resistant infections such as skin infections due to MDR *Mycobacterium abscesses* complex bacteria [30], vancomycin-resistant *Enterococcus faecium* pneumonia [31], MRSA catheter-related bloodstream infection [32], with further *in vitro* research ongoing in relation to antibiotic-resistant organisms including *M*. *tuberculosis* [33–37].

### Combination Drugs

One key area where novel antibiotics have been developed relates to those with the potential to treat antibiotic-resistant Gram-negative organisms [38]. Development of combination drugs has been evident in an attempt to treat difficult and antibiotic-resistant bacteria. Novel β-lactam and β-lactamase combination antibiotics have been an area of recent development and subsequent approval [39].

EMBLAVEO® (Pfizer, AbbVie) is the most recent FDA approved (07 February 2025) and EMA approved combination antibiotic for marketing authorization (22 April 2024). This combination antibiotic consists of the monobactam β-lactam aztreonam and avibactam which is a broad-spectrum β-lactamase inhibitor. This combination has been approved 39 years since the approval of aztreonam. EMBLAVEO® is effective against Gram-negative organisms such as *Escherichia coli, Klebsiella pneumoniae, Klebsiella oxytoca, Enterobacter cloacae complex, Citrobacter freundii complex,* and *Serratia marcescens* and is licensed to treat adult patients with complicated of intra-abdomenal infections, hospital-acquired pneumonia, ventilator-associated pneumonia, urinary tract including pyelonephritis, and aerobic Gram-negative infections which have limited treatment options particularly due to MDR [40]. Another recent antibiotic combination drug is sulbactam–durlobactam (XACDURO®) which is a β-lactam (sulbactam)/β-lactamase inhibitor combination (durlobactam) which was approved in May 2023 for the treatment of infections caused by *Acinetobacter baumannii-calcoaceticus* complex [41]. Cefepime/enmetazobactam (EXBLIFEP®; Advanz Pharma & Allectra Therapeutics) is an example of a novel antibiotic which received accelerated assessment via the MHRA International Recognition Procedure (IRP), resulting in approval within 55 days, on 4 April 2024, for the treatment of severe urinary tract infection and hospital-acquired pneumonia [42] and has potent antibacterial activity against Extended-Spectrum Beta-Lactamase (ESBL) *Enterobacterales* [43].

Several other combination antibiotic drugs developed approved over the last 10 years with a therapeutic indication for Gram-negative infections including imipenem/cilastatin/relebactam (RECARBRIO®; Merck Sharp & Dohme) [44], meropenem/vaborbactam (Vaborem®; Menarini) [45], ceftazidime/avibactam (Avycaz®; AbbVie) and ceftolozane and tazobactam (ZERBAXA®; Merck Sharp & Dohme) [46], the mechanism of action all of which relate to interference with the synthesis of the bacterial cell wall.

The scientific community continues to research combination antibiotics against antibiotic-resistant organisms such as *K*. *pneumoniae* [47]. Additionally, synergy testing of various multiple target antibiotic combinations offers guidance to clinicians when treating antibiotic-resistant organisms [48].

### Drug Repurposing

Drug repurposing has extensive potential to accelerate the development of *de novo* antibiotic therapies and reduce the expense and failure rate for the application of such drugs against MDR bacterial infections, because safety and efficacy data already exist for other therapeutic applications [49]. Various approaches have been used to evaluate the potential antimicrobial repurposed drugs, including virtual screening and computational methods [50]. Recently a high throughput screening method which utilised a spectrophotometric approach prior to primary *in vitro* screening, for growth inhibition and anti-biofilm activity, was used by Pompilio and colleagues in the search for drugs with potential antimicrobial and antibiofilm properties against a MRSA strain from a patient with CF [51]. From this study, it was interesting to note that several antibacterial and antibiofilm compounds conventionally used as diuretic, anti-cancer, anti-asthmatic, anti-histaminic and non-steroidal anti-inflammatory drugs, were identified which warrant further investigation.

Repurposing research has primarily focused on MDR organisms and extended drug-resistant organisms such as *A. baumannii* [52], *P. aeruginosa* [53], *M*. *tuberculosis* and the non-tuberculous mycobacteria, such as *M. abscessus* [54]. A selection of such studies is detailed in Table 1. For a comprehensive appreciation, please see a recent review on the subject area [49]. In general, although research has indicated the potential of many of these proposed repurposed drugs, further research and clinical trials are warranted before such use becomes a reality.

**TABLE 1:** A selection of non-antibacterial drug repurposing studies, evidencing a direct antibacterial or synergistic or restoration effect in the presence of conventional antibiotics.

Class drug	Action	Bacteria
**Antidiabetic**	​	​
Metformin [55]	Quorum quenching, decrease in motility	*P. aeruginosa*
**Anticancer**	​	​
VLX600 [56]	Iron chelator	*Mycobacterium abscessus,* *E. coli, S. aureus, P. aeruginosa*
**Antidepressant**	​	​
Paroxetine [57] Fluoxetine [57]	Inhibition of biofilm formation, synergy with levofloxacin	MDR *P. aeruginosa*
**Antifungal**	​	​
Ciclopirox [58]	Antibacterial and antibiofilm activity	*P. aeruginosa*
**Antihelminthic**	​	​
Albendazole [59]	Inhibition of quorum sensing, anti-virulence, anti-biofilm properties	*P. aeruginosa*
**Antihistamine**	​	​
Ebastine [60]	Bactericidal, antibiofilm, disruption of bacterial membrane/ increasing membrane permeability	*S. aureus*, MRSA
Fexofenadine [61] Levocetrizine [61]	Anti-quorum sensing, antivirulence potential	*P. aeruginosa*
Astemizole [62]	Disrupted bacterial membrane integrity, inhibited ATP synthesis, induced ROS accumulation	MRSA
**Antipsychotic**	​	​
Chlorpromazine [52]	Restoration of susceptibility to ciprofloxacin and levofloxacin Bactericidal activity, efflux pump inhibitor	PDR and MDR *A. baumannii*
Penfluridol [63]	Limited antibacterial activity alone, synergy with colistin. Enhanced outer/inner membrane permeability, inhibition and biofilm eradication	Colistin-resistant *E. coli,* *K. pneumoniae,* *A. baumannii, P. aeruginosa*
**Antiviral**	​	​
Ribavirin [58]	Antibiofilm activity	*P. aeruginosa*
**Beta blocker**	​	​
Propranolol [52]	Restoration of susceptibility to ciprofloxacin and levofloxacin	PDR and MDR *A. baumannii*
**Calcium channel blocker**	​	​
Fendiline [64]	Inhibition of essential lipoprotein trafficking pathways	Carbapenemase expressing *A. baumannii*
Amlodipine [53]	Reduction in biofilm formation	*P. aeruginosa*
**Diuretic**	​	​
Furosemide [58]	Anti-biofilm activity	*P. aeruginosa*
**Immunomodulator**	​	​
Fingolimod [65]	Bactericidal, inhibition of biofilm formation, disruption of cell permeability/integrity	*S. aureus*, MRSA, *E. faecalis, S. agalactiae*
**Nonsteroidal anti-inflammatory**	​	​
Aspirin [66]	Synergistic bactericidal activity with colistin Aspirin-colistin disrupted cell membrane	MDR *P. aeruginosa*
Ibuprofen [67]	Effects intracellular K^+^ flux and leakage resulting in destabilisation of cytoplasmic membrane	*S. aureus*
Diclofenac [68]	Increases oxidative stress and decreases type IV pili when used with colistin resulting in sensitization of resistant strains to colistin	PDR and MDR *A. baumannii*
Aceclofenac [61]	Anti-quorum sensing, antivirulence potential	*P. aeruginosa*
**Statin**	​	​
Atorvastatin [61]	Anti-quorum sensing, antivirulence potential, interference with proton-motive force	*P. aeruginosa*
**Thrombopoietin receptor agonist**	​	​
Eltrombopag [69, 70]	Bacteriostatic, antibiofilm, anti-persister effects	*S. epidermidis,* MRSA
**Veterinary anti-parasitic**	​	​
Nicolaides [71]	Impact bacterial catabolic pathways resulting in reduction of ATP thereby inhibiting bacterial division/growth. Inhibition of α-haemolysin secretion by *S. aureus*	Gram-positive bacteria MRSA, *E. faecalis,* VRE, S. agalactiae, *S. suis*, *S. pneumoniae*
Ivermectin [61]	Anti-quorum sensing, antivirulence potential	*P. aeruginosa*

MDR, multi-drug resistant; MRSA, methicillin-resistant Staphylococcus aureus PDR, pan-drug resistant; VRE, vancomycin-resistant enterococci.

## Novel Antimicrobials in Research and Development

### Antimicrobial Peptides and a Novel Macrocyclic Peptide

Antimicrobial peptides (AMPs), are natural components involved in the innate defence response against pathogenic organisms and are found in plants, animals, amphibians, insects, humans and microorganisms [72]. AMPs have received much attention due to their direct antimicrobial properties as well as their potential modulation of both the innate and adaptive immune responses and regulation of inflammatory processes [73].

AMPs, have broad specificity and are comprised of 5–100 amino acids, typically 50, with a molecular mass of 2–7 kDa [74, 75]. The primary mode of antimicrobial action of these positively charged peptides, is due to their Arginine (Arg) and Lysine (Lys) amino acid residues, which allow for the selection of negatively charged microbial membranes and subsequent disruption of these membranes by means of hydrophobic or electrostatic interactions resulting in cell lysis [74, 75]. Additionally, AMPs may inhibit protein or nucleic acid synthesis, protease activity and bacterial cell division [75]. Duarte-Mata & Salinas-Carmona recently discussed the potential of AMPs for the treatment of intracellular bacteria such as *Mycobacterium tuberculosis* due to the ability of AMPs to kill such organisms by means of internalisation, penetration and the induction of peptides by infected cells and bacterial clearance by means of AMP immunomodulation [73]. This would be a novel focus of research as to date studies and clinical trials have focused on extracellular bacteria.

It has recently been reported that although over 3,000 AMPs have been discovered only seven (gramicidin D, daptomycin, vancomycin, oritavancin, dalbavancin, telavancin and colistin), all of which have originated from soil bacteria, have been approved to date by the FDA [76]. The concerns and limitations of AMPs as a potential therapy must be acknowledged, which may have contributed to their lack of approval. Adverse effects have included kidney injury as well as toxicity namely due to cytotoxic and haemolytic effects. Additionally, historically some AMPs have shown undesirable characteristics with respect to solubility and stability and poor efficacy or non-superiority in comparison to conventional antibiotic therapy [73, 75], which may be in part due to their degradation with blood proteases or by the binding with other proteins. As such consideration must be given to the mechanism of administration [73].

An interesting, novel class of antibiotic with narrow spectrum is a macrocyclic peptide targeting *A*. *baumannii*, is zosurabalpin which is due to enter Phase 3 clinical trials in late 2025/early 2026 [77]. Zosurabalpin, has been identified and optimised as a result of initial *in vitro* studies to elucidate its antibacterial and pharmacokinetic properties and subsequent *in vivo* animal studies. The mechanism of action of this novel class of antibiotic is blocking the transport of lipopolysaccharide from the inner membrane of *A*. *baumannii* to its destination on the bacteria’s outer membrane. [78]. This is of significance as zosurabalpin should not be affected by current known resistance mechanisms.

### Antibiofilm Approaches & Inhibition of Quorum Sensing

The ability of communities of bacteria to form biofilms is problematic for many disease states and infections, e.g., infective endocarditis, lung infections in CF and infections associated with medical devices and implants. The biofilm matrix and intracellular signalling mechanisms, such as quorum sensing, between polymicrobial communities to control biofilm formation, and polymicrobial competition are important aspects to consider in relation to AMR. The composition of the biofilm such as the matrix and in particular the protective extracellular polymeric substances (EPS), coupled with metabolic dormancy of pathogens contained within the biofilm, is conducive to protection and intrinsic tolerance to antimicrobial agents. Polymicrobial communities within a biofilm may also contribute to the development and spread of AMR via horizontal gene transfer (HGT), as well as modulating antibiotic efficacy [79]. As knowledge continues to increase in relation to biofilm formation including at a genetic level and via quorum sensing, various groups have investigated novel anti-infective substances natural and synthetic as well as re-purposed drugs (see Table 1), with respect to antibiofilm activity and inhibition of the quorum sensing and associated modulation of virulence pathways, which do not require the eradication of bacteria [80]. Focus has been primarily associated with WHO priority pathogens including *P. aeruginosa* [80] and MDR *A*. *baumannii* [81].

### Antibacterial Oligonucleotides

Antibacterial oligonucleotides are synthesised nucleic acid sequences designed to exert an inhibitory effect on bacteria by binding to intracellular RNA sequences through complementary base pairing [82]. This technology is based on regulatory gene silencing via antisense RNA which occurs naturally in both prokaryotic and eukaryotic systems. To induce an antimicrobial effect of these oligomers is a result of two mechanisms, namely, by the inhibition of microbial growth by targeting essential gene products [83, 84], or by the inhibition of AMR, by targeting resistance gene products and sensitising pathogens to traditional antibiotics [85–87]. Both applications are viable for combating AMR, the first by providing a new class of antimicrobials, and the second by inhibiting the expression of AMR phenotypes *in vivo*. The antimicrobial efficacy of these molecules is dependent on three key molecular properties; their resistance to degradation, their rate of bacterial cell penetration and their affinity for intracellular target RNAs. Much research in this field has focused on Peptide-conjugated Phosphorodiamidate Morpholino Oligomers (PPMOs) because they have a modified backbone consisting of linked morpholine rings that render them resistant to degradation by nucleases and the oligomer portion specifically binds to mRNA [88]. Membrane-penetrating peptides are conjugated to these oligonucleotides and generally consist of repeating sequence motifs of cationic and nonpolar amino acid residues, which facilitate bacterial uptake, particularly in the case of the membranes of Gram-negative bacteria [88]. PPMOs have been demonstrated to target highly conserved and essential genes such as those coding for acetyl carrier protein (*acpP*), which functions in lipid biosynthesis, and have been found to be highly effective for reducing bacterial load in mouse models of infection for a range of pathogens including *E*. *coli, K*. *pneumoniae, A*. *baumannii* and *P*. *aeruginosa* [83, 84]. Other gene targets include *rpsJ* coding for 30S ribosomal protein S10 whose function is to bind tRNA to the ribosomes and *lpxC* coding for the UDP-(3-O-acyl)-N-acetylglucosamine deacetylase which is involved in lipid A (endotoxin) biosynthesis [88, 89].

Bactericidal PPMOs have also demonstrated anti-biofilm activity both *in vitro* and *in vivo* [83, 84, 88]. In animal models of disease, essential gene targeted PPMOs have not only been shown to inhibit the establishment of biofilm, most likely through growth inhibition of planktonic pathogens, but have also been associated with reduction in mass of previously established biofilm. These findings suggest that despite their large molecular weight, PPMOs can penetrate and act upon biofilms in ways that traditional antibiotics cannot. For AMR-targeted PPMOs, studies have shown effective silencing of transmissible carbapenem resistance genes in *Enterobacteriaceae,* allowing restoration of antimicrobial susceptibility both *in vitro* and *in vivo* [86]. Similar findings of restoring susceptibility to β-lactam antibiotics have been noted for *mecA* mRNA targeted oligonucleotides [85]. mRNA targeting of highly expressed bacterial efflux pumps associated with broad spectrum resistance to fluoroquinolones, tetracyclines, macrolides and β-lactams has also resulted in increased efficacy by reducing minimum inhibitory concentrations for these therapies *in vivo* [87].

To date, PPMOs as antimicrobials have not yet progressed beyond research stages and there are some limitations for antimicrobial applications of this technology that need to be addressed before progression into clinical practice. At present, high concentrations of these high molecular weight molecules are required for effective mRNA silencing *in vivo*, and the potential toxic effect of producing high concentrations of PPMOs systemically needs to be investigated more thoroughly. Another limitation, particularly with reference to resistance gene silencing, lies in the need to determine which AMR phenotype a pathogen is expressing prior to targeted therapy and having to stagger therapy because antibiotic administration prior to achieving an AMR gene silencing effect would be ineffective.

### Nanomaterials

Nanomaterials commonly have at least one dimension or a basic unit in the three-dimensional space in the 1–100 nm range [90]. Nanotechnologies which utilise such nanoparticles (NP) offer several advantages due to their size including improved drug bioavailability due to an increased area of contact between the compound and the bacteria enhancing absorption and adsorption capabilities, and allowing for controlled release and stability [91].

Some NPs which possess hollow structures called nanocages or nanocapsules are designed to contain a drug to deliver and release, and can be made of different materials such as lipids, proteins, polymers, ceramics, silica or metals. It is also possible to use NPs made of materials that already possess antimicrobial activity such as metals, oxides, metal halides or bimetallic materials e.g. ZnO NPs, AgNPs which have demonstrated antibacterial properties against WHO priority pathogens [90].

NPs exert their antimicrobial effects via four main mechanisms; (i) the production of reactive oxygen species (ROS) in the case of metal oxide NPs, which promote peroxidation and damage of the components of the bacterial cell such as polyunsaturated phospholipids in the cell membrane, protein deactivation, enzyme disruption and DNA damage which results in cell death; (ii) physical damage to the cell wall membranes as a result of sharp edges of the nanomaterial; (iii) binding materials on the bacterial cell wall resulting in a loss of the integrity of bacterial cell membranes and the efflux of cytoplasmic substances and (iv) the direct effect of released metal ions which can inhibit ATP production and DNA replication [92, 93].

Surface-functionalised nanocarriers/NPs have been developed with various other functional compounds such as, antimicrobial and antibiofilm compounds such as antibiotics, AMPs, protein, chitosan, ligands, small biomolecules, antibodies and DNA [91] which have shown high antimicrobial activity and synergistic effects against antibiotic resistant bacteria [94] particularly when photodynamic NPs are used [95].

The most frequently studied NPs are silver nanoparticles, AgNPs, which release Ag+ ions and which have a high antimicrobial activity targeting biofilms, the bacterial cell wall and cell membrane, electron transport, signal transduction and generating ROS which target DNA and proteins [96]. Due to these antimicrobial properties, such NPs can be adopted for anti-biofilm coating in the production of surgical implants e.g., in orthopaedics [97] or in the preparation of antimicrobial wound dressings which also promote healing [98]. Kalantari and colleagues highlight some concerns in relation to (i) toxic effects of Ag-NPs in terms of both environmental organisms and human health and (ii) the development of bacteria developing reduced susceptibility/resistance to Ag-NPs [98].

Further research is required to address the challenges relating to production of metal NPs such as Ag-NP in a safe, environmentally friendly and cost-effective manner, in addition to addressing the varied reports relating cytotoxic and genotoxic effects of some metal-NPs such as Ag-NP, TiO_2_-NP [99].

## Natural Sources of Novel Antibiotics From the Environment

### Soil

The soil is a rich source of bacteria and fungi which produce antibacterial and antifungal compounds and historically researchers have searched the soil microbiota in the goal to find such compounds, particularly those which have an antibacterial action to help in the drive against AMR [100]. One limitation, however, has been that not all such environmental organisms are culturable by routine culture media and incubation conditions. In order to address this limitation, iChip technology was developed, whereby environmental samples containing micro-organisms were placed in micro-chambers and subsequently placed into their natural environment for incubation [101]. Using such technology lead to the discovery of an uncultured bacterium and novel antibiotic, teixobactin [102]. Teixobactin, is a new class of antibiotic which has a dual action, namely inhibition of cell wall synthesis by binding to a highly conserved motif of lipid II (precursor of peptidoglycan), thereby inhibiting peptidoglycan synthesis as well as disruption of the cytoplasmic membrane [102]. Much interest and research has been conducted on this antibiotic as it only damages membranes which contain lipid II which negates toxicity in human cells. Also of note is that this antibiotic has shown minimal resistance [103].

More recently, an environmental bacterium *Paenibacillus* sp. was shown to exert a broad-spectrum antibacterial activity. Examination of this bacterial genome revealed the presence of a biosynthetic gene cluster (BGC) of colistin and interestingly a BGC of a lasso peptide subsequently named, lariocidin (LAR) [104]. Lasso peptides, so called due to their structural knotted lasso shape, belong to the class of peptides which are synthesised ribosomally and are subsequently modified post-translationally RiPPs) [104]. LAR is of major interest due to several reasons, as it (i) is the first lasso peptide that targets the ribosome to interfere with protein synthesis, specifically by binding at a unique site in the small ribosomal subunit and interacting with the 16S rRNA and aminoacyl-tRNA, to inhibit translocation and induce miscoding, (ii) has low propensity mutations spontaneous resistance mechanisms; (iii) lacks toxicity towards human cells and (iv) has potent broad-spectrum antibacterial activity against organisms including *A*. *baumannii* [104, 105]

### Ethanopharmacology

Phytochemicals, which are bioactive compounds from plants and numerous research studies have documented that these are a source of antimicrobial natural compounds due to their broad spectrum of activity both *per se*, as adjuvants and synergistic compounds, enhancing the activity of conventional antibiotics [106–108]. Chinese herbal medicine has shown potential in the treatment of infections including antibiotic resistant infections [108, 109]. Although the precise mechanisms of action are difficult to elucidate as approximately fifty herbs in different combinations are employed, research has identified in the case of coumarins, the blocking of anti-quorum sensing and biofilm formation e.g. [110] and efflux pump inhibition [108, 111].

### Honey

Honey, in particular Manuka honey, has had a rich history of being used to treat infections particularly in relation to wound infections [112], with several clinical trials currently ongoing for the treatment of wounds such as deep neck abscesses (NCT06562257), burn wounds (NCT03674151) and recent research has focused on the potential antibiotic effects of honey against antibiotic resistant pathogens both in human and veterinary medicine [113–115]. The antibiotic properties of honey have been attributed to its physicochemical properties, such as low pH, low water content and high osmolarity, as well as its composition of components including hydrogen peroxide, methylglyoxal (MGO), particularly in the case of Munuka honey from the Australian bush *Leptospermum* sp [116]. and defensin-1, as well as secondary metabolites originated from nectars, such as flavonoids and phenolic compounds [117–119]. The composition of honey varies depending on the botanical source, species of bee, geographical region and the microbiome of raw honey contributing to its physicochemical properties [120].

**FIGURE 3:**
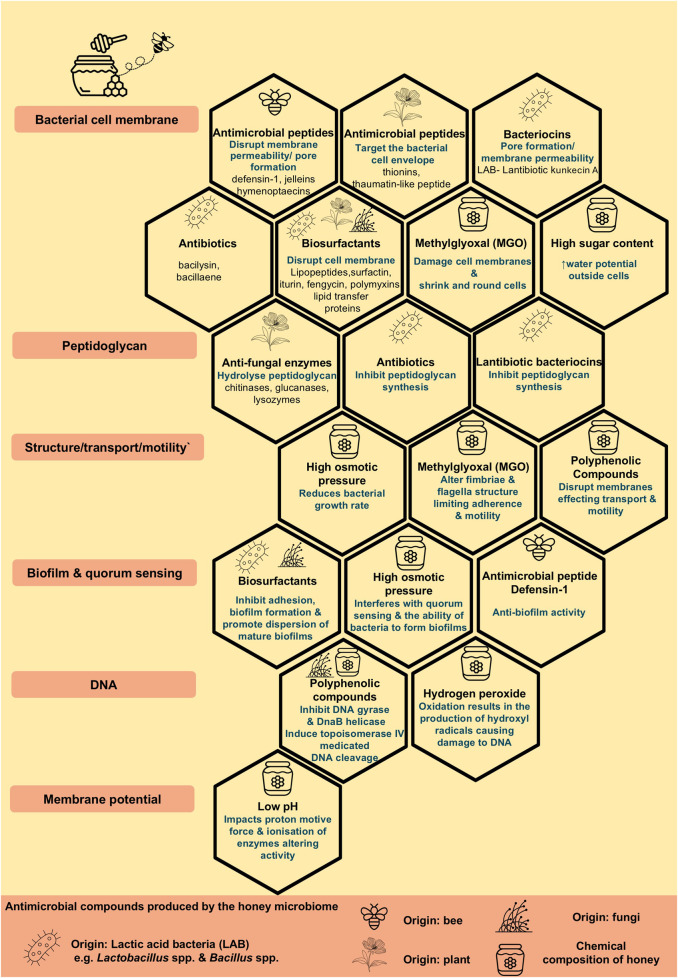
The composition of honey and antimicrobial compounds produced by the honey microbiome contributing to the antimicrobial properties of honey.

It must be realised however, that the antimicrobial effects shown by honey are not necessarily attributed to one particular compound and currently research is focusing on elucidating the mechanism of action of the diverse antimicrobial compounds found in honey many of which have been sourced from the bee, the plant/nectar and the associated microbiomes and microbial interactions [118]. A recent review by Brudzynski, 2021 [118] provides an interesting overview of the microbial ecosystem and the various antimicrobial compounds produced by the microbiome of honey, the honeybee, originating plants, bacteria and fungi. Such antibacterial compounds include ribosomal peptides, non-ribosomal peptides (NRP) peptides, namely antibiotics, lipopeptide surfactants, siderophores and polyketides from *Bacillus* sp. and bacteriocins and autolysins from lactobacilli (Figure 3) MGO has recently been incorporated into a novel liposomal formulation containing tobramycin which has shown active reduction in biofilm formation, as well as inhibition of bacterial adhesion highlighting the therapeutic antimicrobial potential of its components [121].

### Spices and Essential Oils

Various spices have been examined for their therapeutic properties, antimicrobial activity and mechanisms of action [122], as well as their adjuvant activity in conjunction with conventional antibiotics against drug resistant organisms e.g., *M. abscessus* [123], polymyxin-resistant *Klebsiella aerogenes* [124] and *P*. *aeruginosa* [125] and Gram-negative organisms causing urinary tract infections [126]. Research has focused on not only pathogens which impact human health but also on the properties of spices which contribute to the prevention of foodborne pathogens, food safety and food preservation [127].

### Venom

The potential antibacterial properties of venom from various sources have been shown against drug and MDR pathogenic bacteria. Most recently, honeybee venom has been demonstrated to be active against MDR pathogenic bacteria including, *E. coli, Salmonella Typhimurium, *and *Enterococcus faecalis* [128] and *S*. *aureus* [129]. Spider venoms have also been shown to contain a valuable resource of antimicrobial peptide toxins against pathogens such as *S. aureus* in the case of the Lynx spider *Oxyopes forcipiformis* [130]. The antibiotic potential of venoms including antimicrobial peptides sourced from jellyfish [131], scorpions [132, 133], wasps [134] and insects and centipedes [135], anti-biofilm substances from snake venom [136, 137] and antimycobacterial peptides from the venom gland of the cone snail *Conasprella ximenes* [138], have provided an evidence-base for future research into the search and development of novel antibacterial pharmacological agents.

### Extreme Environments

Bioprospecting of extreme environments has identified various sources of antimicrobials [139]. Below a selection of examples show that all areas of earth are being explored in the quest to discover sources of novel antimicrobial compounds.

Antarctica, has been described as the coldest region on earth by NASA, where the hollows in the high ridge of the East Antarctic Plateau have recorded air temperatures of −94 °C and minimum surface temperatures of −98 °C [140] and yet it has been recognised as a valuable source of novel antimicrobials following microbial ecology [141] and genome mining [142, 143]. Indeed, potential therapeutic value of these novel antimicrobial compounds and source organisms, including bacteria, fungi, lichen, fish, seaweeds, sponges, krill, penguin and springtail have been recognised resulting in an increasing number of patents [144]. Antarctic fish/ice fish such as *Notothenia coriiceps*, *Parachaenichthys charcoti*, *Trematomus bernacchii* and *Chionodraco hamatus*, have been shown as a source of piscidins, which are antimicrobial peptides, with activity against particularly Gram-negative bacteria and MDR bacteria [145–149]. Other bacterial sources of novel, bioactive compounds, have been sourced from symbiotic bacteria colonised on Antarctic fish [150] and bacteria found in Antarctic marine soils [151, 152], sediment [153, 154], and Antarctica marine water [155] and fungi found in lichen [156] A recent study of interest showed supernatants from several Antarctic marine bacteria, whilst not antimicrobial *per se*, prevented biofilm formation and dispersal of biofilms produced by ESKAPE pathogens (*Enterococcus faecium, Staphylococcus aureus, Klebsiella pneumoniae, Acinetobacter baumannii, Pseudomonas aeruginosa, and Enterobacter* spp.) [154]. Other antibiofilm proteins produced by Antarctic bacteria such as *Pseudomonas* spp. TAE608 [152] and *Psychrobacter* sp. TAE2020 [155] have also been reported.

Bioprospecting has led to the discovery of actinobacteria, with antimicrobial activity in the Kubuqi desert in China [157]. *Streptomyces* spp. found in the Saharan desert soils have been shown to produce a novel broad-spectrum antimicrobial which is a hydroxamic acid-containing molecule, with antagonistic properties against MDR pathogens [158]. The soil, in other remote areas such as caves in China, have been shown to comprise of *Streptomyces* spp. which produce xiakemycin A, which is a novel pyranonaphthoquinone antibiotic, with a strong inhibitory action against Gram-positive bacteria [159]. A wealth of research is ongoing in sourcing antimicrobials obtained from global extreme environments, such as nanoparticles from volcanic silica [160]; antibacterial activities and compounds including those which inhibit biofilm formation, from extremophile bacterial [161] and fungal [162] microorganisms, as well as shrimps [163] within deep-sea hydrothermal vents and systems. The high-altitude region in the Andes, namely the Lirima hydrothermal system, located in the northern region of Chile has been shown to be a source of secondary metabolites with antimicrobial activity produced by thermophilic bacteria. [164]. The halophilic environment is also being explored as a potential source of novel antimicrobial agents [165], with a recent study reporting that extracts from the soil from the Dead Sea in Jordan, had an antibiofilm activity against *P*. *aeruginosa, E*. *coli*, and *S*. *aureus* isolated from diabetic patients’ ulcerated wound infections of the feet [166]. The antimicrobial activity of the Dead Sea soil extract exerted a multifactorial action in that it (i) inhibited biofilm formation by reducing the production of extracellular substances and alginate; (ii) negatively impacted bacterial adhesion by decreasing surface hydrophobicity; (iii) disrupted preformed biofilms and (iv) disrupted outer bacterial membranes [166].

## Phage Therapy

### Clinical Application of Phage Therapy

Bacteriophages, or phages for short, are viruses which infect a bacterium and undertake a lysogenic or lytic pathway and can potentially transfer genetic material such as virulence and antibacterial resistance genes and lyse bacteria, respectively [167]. Bacteriophages have a number of characteristics which make them advantageous candidates to treat antibacterial resistant infections, such as their specificity in relation to bacterial hosts, the ability to cause bacterial death and their ubiquitous nature in that they are found in the environment as well as animals and humans, where the human gut phagosome has been shown to play a role in gut health and human disease. [167]. Furthermore, due to their natural existence within the human microbiome, it is assumed that using such bacteriophages for therapeutic purposes could be conducted safely and efficiently [168].

Of particular interest is the real-world application of phage therapy to treat or suppress infection. Within the scientific literature, generally such reports are confined to individual case studies and small cohorts, although there are several clinical trials and larger studies documented some of which are ongoing (Table 2), however valuable lessons can be learnt from such studies. The first reported clinical use of phage therapy to treat an infection was in 2017 in USA, when a multidrug-resistant *A*. *baumannii* infected pancreatic pseudocyst in a diabetic patient with a necrotising pancreatitis was successful, following a nine-phage cocktail administered intravenously and percutaneously into the abscess cavities [169]. This group subsequently established the Center for Innovative Phage Applications and Therapeutics (IPATH), University of California, San Diego and published details of the outcomes of requests and ten cases which underwent intravenous phage therapy, in combination with systemic antibiotics, due to MDR and antibiotic-recalcitrant infections. The ten cases related to various infections due to *S. aureus* (n=2), *E. coli* (n=1), *A. baumannii* (n=2) and *P. aeruginosa* (n=5) [170]. The preferred route of administration was intravenous although one patient with pneumonia due to *P. aeruginosa* additionally received nebulised phage therapy and where possible a cocktail of phages was used to minimise the development of phage-resistance. The authors reported that such phage therapy was safe and following the initial administration at clinic, patients were administered their phage therapy at home. Furthermore, such phage therapy was not only successful as a treatment but as a suppressive therapy [170]. It is important to note that although bacterial resistance occurred in 3/10 patients, this was able to be successfully overcome by introducing additional phages which had matched with the resistant bacterial isolates.

**TABLE 2:** Ongoing and recently completed clinical trials, within the last 5 years as of May 2025, relating to bacteriophage therapy to treat infections.

Clinical Trials.gov ID	Condition	Phase	Status	Enrolment	Start date	Completion/ estimated date	Country
NCT06605651	Hip or knee prosthetic joint infections due to *Staphylococcus aureus*	2	Not yet recruiting	100	01/2025	01/2027	Unknown
NCT06814756	*Morganella morganii* prosthetic joint infection	1/2	Not yet recruiting	1	24/02/2025	06/2026	Canada
NCT06750588	Acute alcohol-associated hepatitis (*E. faecalis*)	1	Not yet recruiting	12	01/03/2025	12/2025	USA
NCT06409819	Recurrent urinary tract infections in kidney transplant recipients	1/ 2	Not yet recruiting	32	01/06/2024	30/06/2027	USA
NCT06942624	Chronic *Enterococcus faecium* periprosthetic joint infection	1/2	Not yet recruiting	1	05/2025	06/2026	Canada
NCT05590195	Urinary and vaginal health	3	Not yet recruiting	50	01/05/2024	01/06/2025	UK
NCT06370598	Ventilator-associated pneumonia	1/2	Not yet recruiting	15	09/2024	06/2025	France
NCT06262282	People with cystic fibrosis and non-tuberculosis mycobacteria pulmonary disease	Observational	Enrolling by invitation	10	05/02/2024	12/2028	USA
NCT05314426	Mayo clinic phage program biobank	Patient registry	Enrolling by invitation	100	19/04/2022	04/2027	USA
NCT06938867	Patients scheduled for allogeneic hematopoietic stem-cell transplantation receiving fluoroquinolone prophylaxis and harbouring fluoroquinolone-resistant *Escherichia coli* pre-transplant	1/2	Recruiting	240	25/02/2025	01/04/2026	USA
NCT06559618	Spinal cord injury patients with bacteriuria	1	Recruiting	30	02/03/2025	12/2026	USA
NCT05967130	Chronic urinary tract infection post kidney transplant	3	Recruiting	20	01/07/2023	01/07/2027	Islamic Republic of Iran
NCT06870409	Infective endocarditis	3	Recruiting	30	05/02/2025	05/02/2029	Russian Federation
NCT06185920	Severe infections	Observational	Recruiting	250	01/02/2023	01/02/2033	France
NCT06319235	Surgical site infections caused by *Staphylococcus aureus* and *Pseudomonas aeruginosa*	1/2	Recruiting	52	27/10/2023	31/12/2025	Czechia
NCT04724603	Phage safety retrospective cohort study	Observational	Recruiting	25	01/02/2021	01/08/2022	France
NCT05369104	Prosthetic joint infection due to *Staphylococcus aureus*	2	Recruiting	64	15/06/2022	16/06/2025	France
NCT04650607	Phage safety in treating prosthetic joint or severe infections	Observational	Recruiting	100	09/05/2022	09/05/2028	France
NCT06368388	Difficult-to-treat infections	Observational	Recruiting	50	01/06/2021	01/06/2025	Belgium
NCT05177107	Diabetic foot osteomyelitis	2	Recruiting	126	24/11/2021	12/2024	USA
NCT05948592	Diabetic foot infection	2	Recruiting	80	08/11/2023	31/12/2024	USA/ India
NCT05488340	Uncomplicated urinary tract infection caused by drug resistant *E. coli*	2	Recruiting	318	13/07/2022	12/2025	USA
NCT06456424	Methicillin-sensitive *Staphylococcus aureus* prosthetic joint infection	1/2	Active, not recruiting	1	20/11/2024	11/2025	Canada
NCT05182749	Shigellosis	1/2	Active, not recruiting	52	23/02/2023	30/06/2025	USA
NCT05537519	Urinary tract infection	1/2	Active, not recruiting	1	01/05/2023	30/06/2024	Canada
NCT06827041	Periprosthetic joint infection	1	Active, not recruiting	1	22/02/2024	02/2025	Canada
NCT05010577	Cystic fibrosis patients with chronic *Pseudomonas aeruginosa* pulmonary infection	1/ 2	Active, not recruiting	32	21/06/2022	03/2024	USA/ Czechia/ Israel/ Netherlands/ Spain
NCT04682964	Tonsillitis	3	Active, not recruiting	128	02/10/2020	31/12/2028	Uzbekistan
NCT06798168	Periprosthetic joint infection of multidrug resistant *Pseudomonas aeruginosa*	Expanded access	Available	-	-	-	Canada
NCT05453578	Cystic fibrosis individuals chronically colonized with *Pseudomonas aeruginosa*	1/2	Completed	72	03/10/2025	10/04/2025	USA
NCT05184764	Bacteraemia due *to Staphylococcus aureus*	1/2	Completed	50	26/24/2022	14/01/2025	USA/ Australia
NCT05616221	Subjects with non-cystic fibrosis bronchiectasis and chronic pulmonary *Pseudomonas aeruginosa* infection	2	Completed	48	10/01/2023	17/07/2024	USA
NCT04684641	Cystic fibrosis subjects with *Pseudomonas aeruginosa*	1/2	Completed	8	29/03/2021	26/05/2023	USA
NCT04325685	Effect of supraglottic and oropharyngeal decontamination on the incidence of ventilator-associated pneumonia and associated microbiomes	N/A	Completed	60	01/01/2020	01/11/2023	Russian Federation
NCT04596319	Subjects with cystic fibrosis and chronic pulmonary *Pseudomonas aeruginosa* infection	1/2	Completed	29	22/12/2020	14/12/2022	USA
NCT05498363	Difficult-to-treat infections	Observational	Completed	100	01/01/2008	31/12/2021	Belgium
NCT04803708	Diabetic foot ulcers	1/2	Completed	20	22/03/2021	07/08/2022	Israel
NCT04191148	Lower urinary tract colonization caused by *Escherichia coli*	1	Completed	36	30/12/2019	19/11/2020	USA
NCT04323475	Wound infections in burned patients	1	Unknown	12	01/2022	08/2023	Australia
NCT02664740	Diabetic foot ulcers infected by *Staphylococcus aureus*	1/2	Unknown	60	01/06/2022	08/2024	France
NCT04815798	Prevention and treatment *of Staphylococcus aureus*, *Pseudomonas aeruginosa*, and *Klebsiella pneumoniae* colonized pressure injuries	1/2	Unknown	69	01/2022	12/2023	USA

Although clinical successes have been noted with respect to phages in combination with systemic antibiotic therapy, there are ongoing *in vivo* studies investigating the mechanisms involved which could potentially lead to treatment success or failure, the findings of which have been conflicting. A recent article by Khosravi et al. serves as a critical evaluation of the phage therapy in an attempt to promote adjuvant therapy particularly in the case of individuals with chronic lung infections, such as those with CF, chronic pulmonary disease, non-CF bronchiectasis and individuals with chronic rejection following lung transplantation [171]. Khosvravi et al. details evidence to suggest a combination therapeutic approach can have a synergistic effect and that bacteria can be re-sensitised to antibiotics. In contrast, there have been studies evidencing phage-antibiotic antagonism in the form of phage resistance and enhanced antibiotic resistance, as such it has been proposed that phage therapy and antibiotic therapy should be staggered to minimise such resistance [171].

### Is Phage Therapy for Difficult Infections a Reality Within the UK?

Due to the small number of case studies and limited clinical trials, in 2023, NHS Scotland considered a report from the Scottish Health Technologies Group (SHTG), in relation to “Bacteriophage therapy for patients with difficult to treat infections” [172]. The SHTG critically evaluated the evidence from the scientific literature and concluded that although there was a limited evidence-base relating to safety and clinical effectiveness of such therapy, and the lack of large-scale clinical trials, such therapeutic approaches have proven effective in individuals with infections which are difficult to treat with conventional antibiotics. Due to the lack of published cost-effectiveness studies, the SHTG undertook an economic modelling approach to evaluate the potential clinical use of phage treatment in conjunction with conventional care of refractory diabetic foot infections in individuals who were at high risk of lower extremity amputation and concluded this to be of a potentially cost-effective application of phage therapy. It was also recommended that the use of phage therapy in Scotland should be evaluated in terms of clinical effectiveness and safety to further inform decisions in future applications of phage therapy [172].

More recently, the House of Commons, UK Parliament, published a Committee report on 3 January 2024, by the Science, Innovation and Technology Committee relating to “The antimicrobial potential of bacteriophages”. The committee considered global witness from academia, clinicians, regulators, government officials and funding bodies in the format of oral presentations and written evidence on the safety, efficacy, manufacturing of phages, phage clinical trials and clinical use within the UK to date as well as evidence from global witnesses and site visits [173]. In summary, the House of Commons Science, Innovation and Technology Committee made eighteen recommendations, which comprised of four themes relating to phages namely, safety, efficacy and the UK phage research base, manufacturing of phages, clinical trials and the clinical use in the UK [173]. Subsequently, on the 1 March 2024, the UK government responded to these recommendations and the full policy paper can be viewed at the government website [174]. In summary, although the UK government accepted that the current evidence-base of phage therapy was promising, they believed that further evidence would be necessary to gain a full understanding of how such therapy could aid in combating AMR and that they would continue to work and support appropriate partners to achieve this aim. The UK government also indicated that phage therapy would be included amongst a range of various research areas in consideration for the treatment of AMR infections both in animals and humans, and the continued evidence will be reviewed and considered. They also highlighted what they considered to be the current limitations relating to the deployment of phage therapy in the UK which primarily related to “quality assurance, supply chain adequacy, financial approvals, health, safety and containment, and usage guidelines” [174]. A potential roadmap for the deployment of phage therapy within the UK has not been proposed by the UK government, however Jones et al. have proposed such guidance, particularly in relation to scalability and Good Manufacturing Practice (GMP) [175].

To date, phage therapy, is classified as a biological medicine, and is not licensed by the MHRA in the UK. Jones et al. discuss the regulatory situation in relation to phage therapy within in the UK and in short, phages may be used as unlicensed medicinal products also known as “*specials*” or “*named patient*” alternatives in accordance with MHRA guidance when conventional treatments are refractive, and the clinician deems an alternative therapeutic intervention is required [175]. If phages are imported for such clinical purposes, there is no requirement to be manufactured according to GMP, however, MHRA guidance must be adhered to. Phages manufactured in the UK for the purposes of clinical or investigational use must be manufactured according to GMP [175].

## Antimicrobial Photodynamic Therapy (aPDT)

Light emitted at a precise wavelength in association with a photosensitizer (PS), can generate lethal photo-oxidative stress by producing detrimental forms of oxygen as radicals or reactive oxygen species (ROS), resulting in damage to cellular structures, such as membrane structure, and other components of pathogens such as DNA, cytoplasmic membrane proteins and lipids. Such damage alters cell wall synthesis, damages virulence factors and prevents replication and DNA synthesis [176]. Two types of photochemical reactions, result from activation of photosensitisers, namely Type I which result from the transfer of radical ions (such as superoxide anions (O_2_^−^), leading to the formation of various free radicals (including hydroxyl radicals HO˙, peroxyl radicals ROO˙ and alkoxyl radicals RO˙) and radical ions (radical cation of thymine or guanine) and Type II reactions which result in reactive singlet oxygen species, with both reaction types targeting various pathogen biomolecules [177].

To date there has been research into the application of this approach in relation to the treatment of skin cancers; however, there has also been research into the potential bactericidal and bacteriostatic role of antimicrobial photodynamic therapy (aPDT) in tackling ESKAPE pathogens and the AMR problem in the case of both prokaryotes and eukaryotes [178]. Piksa et al. have reported on studies, primarily *in vitro*, which have used the most common light source, methylene blue in the case of aPDT in relation to Gram-positive, (primarily *S*. *aureus, E*. *faecalis, Streptococcus mutans*), Gram-negative (primarily *E*. *coli, P*. *aeruginosa, Porphyromonas gingivalis*), fungal targets (primarily *Candida* species such as *C. albicans, C. krusei, C. parapsilosis*), as well as viral and parasitic targets [179]. Although not routinely used in clinical practice, primarily due to limitations of using this technology such as a lack of standardisation of protocols, varying light sources and the varied effectiveness of this approach, as of 2022 there were approximately 200 clinical trials using aPDT highlighting the interest and therapeutic potential of this technology [180], with current interest primarily relating to periodontal disease [181]. Table 3 details the most recent clinical trials which are examining the prevention of infection by nasal decolonisation, disinfection, as well as wound healing and tissue repair in the diabetic foot. Research has recently focused on areas which are central to aPDT, namely the depth of penetration and effectiveness of various light sources without resulting in thermal issues, such as laser, light emitting diodes (LEDs), lamp and non-coherent light sources, irradiance and radiance exposure values and also various photosensitizers, including those of both synthetic and natural origin, to ensure high tissue selectivity and that pathogens are selectively damaged rather than host cells [176]. Such research is important so that the potential of aPDT can be realised using light sources which are simple and cost-effective, yet clinically effective [179].

**TABLE 3:** Ongoing and recently completed clinical trials, within the last 5 years as of May 2025, relating to antimicrobial photodynamic therapy (aPDT) to treat and prevent bacterial infections.

Clinical Trials.gov ID	Condition	Phase	Status	Enrolment	Start date	Completion/ estimated date	Country
NCT06777511	aPDT to prevent infection in osseointegrated prosthesis patients	Observational	Not yet recruiting	10	01/08/2025	30/08/2026	USA
NCT06867458	Nasal decolonization using aPDT on the prevention of hospital-acquired pneumonia, ventilator-acquired pneumonia and hospital-acquired bloodstream infection	N/A	Not yet recruiting	400	31/03/2025	04/08/2025	Canada
NCT06731881	The efficacy of PDT for preventing surgical site infections in nasal surgery patients: a pilot study	N/A	Not yet recruiting	80	01/01/2025	31/08/2025	UK
NCT06570252	Investigation of aPDT for preoperative nasal cavity decolonization in adult patients	N/A	Not yet recruiting	208	10/2024	08/2026	Switzerland
NCT06331442	The effect of PDT on accumulation and bacteriological composition of dental plaque in orthodontic patients	N/A	Not yet recruiting	50	05/2024	11/2024	Croatia
NCT06702878	Nasal antimicrobial photodisinfection for the prevention of surgical site infections	3	Recruiting	4514	27/12/2024	07/2025	USA
NCT06416462	Action of aPDT on wound quality and tissue repair in the diabetic foot	N/A	Recruiting	90	30/07/2024	31/06/2026	Brazil
NCT05361590	Impact of regular home use of lumoral dual-light photodynamic therapy on plaque control and gingival health	N/A	Completed	40	11/10/2022	16/08/2023	Finland
NCT06634745	Evaluation of the effectiveness of aPDT using different irrigation activation techniques in teeth with apical periodontitis	N/A	Completed	60	20/06/2023	14/07/2024	Turkey
NCT05797818	The effect of red light photobiomodulation and topical disinfectants on the nasal microbiome	1/ 2	Completed	28	10/01/2023	08/02/2023	USA
NCT05090657	aPDT for nasal disinfection in all patients (universal) presenting for surgery at an acute care hospital for a wide range of surgical procedures	2	Completed	322	04/02/2022	06/08/2022	USA
NCT04047914	aPDT in the nasal decolonization of maintenance haemodialysis patients	N/A	Completed	34	01/11/2019	12/07/2021	Brazil

**FIGURE 4:**
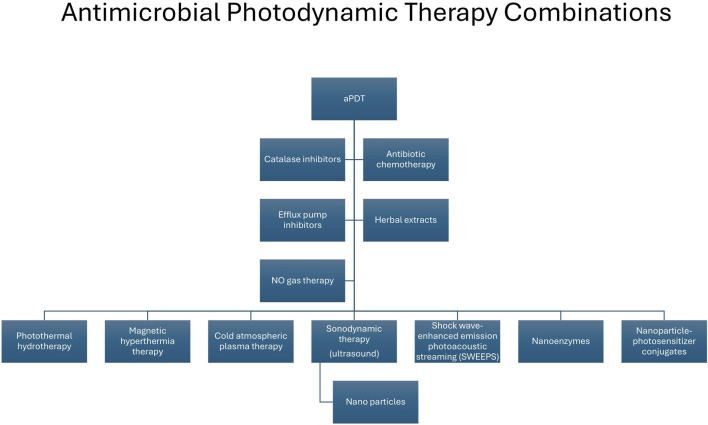
Combination approaches used in conjunction with antimicrobial photodynamic therapy to enhance antimicrobial activity. aPDT, antimicrobial photodynamic therapy; NO, nitric oxide.

aPDT can offer several advantages in that it alone (i) can cause an antibacterial effect in the case of planktonic cells as well as targeting biofilms, (ii) offers limited development of resistance, due to the multiple sites and its mode of action; (iii) is effective against a broad range of pathogens, including MDR bacteria; (iv) has no toxicity, (v) is limited to target cells, (vi) can result in the reduction of virulence factors and pathogenicity and (vii) can result in a potential synergistic effect when used in conjunction with conventional antibiotic therapy and in combination with other therapies (Figure 4; [178, 181]). It must also be acknowledged that there are also some limitations to aPDT therapy including, (i) cost, (ii) weak antibacterial activity in the case of Gram-negative bacteria, (iii) solubility and (iv) specificity [176].

Recently several studies have examined the effectiveness of aPDT against difficult-to-treat and MDR organisms [182] e.g. carbapenemase-producing *K*. *pneumoniae* [183]. Combination of aPDT with antibiotics have shown favourable and even synergistic results in the case of MDR organisms such as *P*. *aeruginosa* and *S*. *aureus* [184], with recent examples, including aPDT in combination with (i) gentamicin and imipenem in *P*. *aeruginosa* isolates [185], (ii) colistin against pan-drug resistant *A*. *baumannii* isolated from a patient with burns [186] and (iii) vancomycin against resistant *E. faecium* [187]. Other drug combinations currently undergoing research with aPDT include efflux pump inhibitors [188] highlighted by two recent studies, the first of which demonstrated an improvement in photo-deactivation of *E. coli* when the efflux pump inhibitor reserpine was used with methylene blue attached to a silver nanoparticle carrier [189]. The second study which used erythrosine B in conjunction with the efflux pump inhibitor verapamil and observed an augmentation effect in the inactivation of MDR planktonic strains of *A*. *baumannii* [190]. It has also been shown that aPDT in combination with quorum-sensing inhibitors resulted in synergistically inhibiting and dispersing the biofilm produced by MRSA [178] and a synergistic effect against *S. aureus* when used in combination with catalase inhibition [191].

It must be noted that several of these physicochemical combinations can be used in isolation or in combination with other therapies and nanoplatforms as antimicrobial approaches. For example, antimicrobial sonodynamic therapy (aSDT) uses low intensity ultrasound waves which can penetrate further than aPDT to a depth of 10 cm in soft tissues, to excite sonosensitizers to generate cytotoxic reactive species that are toxic to pathogens and acts by generating ROS, mechanical pressure and thermal effects [192]. aSDT alone and in combination approach with contrast microbubbles, has proven effective in inactivating both Gram-negative and Gram-positive organisms [193], as well as enhancing antibiotic efficacy in the case of antibiotic-resistant bacteria [194] and eliminating biofilms [194]. Furthermore, a recent study demonstrated that a co-ordination polymer nanoparticle (chlorin e6 (Ce6) with an antimicrobial peptide) in combination with aSDT had the ability to eradicate bacteria as well as exert an eradication of biofilm in the case of MDR-*P. aeruginosa* [195].

## Nitric Oxide (NO)

Naturally, endogenous diatomic free radical nitric oxide (NO), is produced by the first-line innate immune response to invading pathogens. During oxidative bursts, inducible nitric oxide synthase (iNOS) enzymes in macrophages and neutrophils, facilitate the production of NO which subsequently results in the destruction of pathogens within phagosomes due to disruption of protein enzymes required for cell function, modification of membrane proteins and disruption of DNA via deamination.

Due to the multiple mechanisms of action of NO including (i) alternation of microbial DNA, (ii) inhibition of enzymes, (iii) modification of protein targets, (iv) damage to bacterial cell walls, cytoplasmic membranes and the outer membrane of Gram-negative bacteria and (v) dispersal of biofilms, the development of resistance is difficult [196]. As such, its antimicrobial properties make it a valuable antimicrobial agent against infectious agents, particularly MDR bacteria. Although the antimicrobial properties of endogenous NO are well established, Webster and Shepherd (2024) have provided an interesting postulation and cautionary note in relation to the development of novel antibiotics, as they debate that NO may diminish the efficacy of some antibiotics or counteract antimicrobials which target bacterial energetics and elevate metabolism and bioenergetics as part of their bactericidal mechanism, yet they acknowledge evidence for the enhanced lethality of antibiotics in the case of biofilms, when used in conjunction with NO [197].

The off-label use of exogenous inhaled NO gas has been investigated for the treatment of respiratory infections in individuals with CF, which are commonly infected with multidrug resistant organisms [198], including *P*. *aeruginosa* [199], *M*. *abscessus* [200] and *Burkholderia multivorans* [201], as well as individuals with nontuberculous mycobacteria (NTM) pulmonary disease [202, 203]. Results were varied, ranging from reduction in colony forming units, improved lung function, improved antibiotic efficacy and a reduction in biofilm aggregates in the case of *P. aeruginosa* to improved quality of life and lung function, reduction in bacterial load but no eradication in the case of *M*. *abscessus* [196] and an improved antimicrobial susceptibility and clinical outcomes in the case of *B*. *multivorans* [201]. Due to the high reactivity and short half-life of NO (1–5 s) NO donors have been developed as well as delivery systems including nanoparticles, which have shown antibacterial and antibiofilm properties, however there are several areas which require further research in relation to potential toxicity issues, mechanisms to ensure controlled release as well as the optimising penetration of biofilms before its clinical use can be fully investigated [204].

A recent comprehensive article on the antimicrobial effects of nitric oxide by Okda et al. details further *in vitro* and *in vivo* studies as well as case studies, pilot studies, retrospective studies and clinical trials in relation to the potential and real-world therapeutic application in humans [196] and see Table 4 for current ongoing and recently completed trials.

**TABLE 4:** Ongoing and recently completed clinical trials, within the last 5 years, as of May 2025, relating to nitric oxide therapy to treat infections.

Intervention	Clinical Trials.gov ID	Condition	Phase	Status	Enrolment	Start date	Completion date	Country
Inhaled nitric oxide	NCT06950294	Critically ill patients with pneumonia	1	Not yet recruiting	34	06/2025	10/2026	USA
NOX1416; foam based gaseous nitric oxide	NCT06402565	Chronic non-healing diabetic foot ulcers	1	Recruiting	40	25/03/25	30/01/26	USA
Inhaled nitric oxide	NCT06261827	Prevention of nosocomial pneumonia after cardiac surgery	N/A	Recruiting	160	20/02/2024	01/09/2025	Russian Federation
Inhaled nitric oxide	NCT06170372	Nosocomial & community acquired pneumonia	N/A	Recruiting	200	15/02/2024	15/01/2026	Russian Federation
Nitric oxide releasing solution (nasal spray)	NCT06264141	Recurrent acute bacterial rhinosinusitis	2	Active, not recruiting	162	16/01/2024	24/02/2025	Bahrain
Inhaled nitric oxide agent, RESP301, via nebulisation	NCT06041919	Adults with rifampicin susceptible tuberculosis	2	Active, not recruiting	75	27/09/2023	31/07/2025	South Africa
Inhaled nitric oxide	NCT06162455	Prevention of nosocomial pneumonia after cardiac surgery	N/A	Completed	74	17/11/2023	15/01/2024	Russian Federation
Nitric oxide releasing solution	NCT04755647	Diabetic foot ulcer	1/2	Completed	40	23/02/2021	20/05/2023	Canada
Intermittent inhaled nitric oxide	NCT04685720	Nontuberculous mycobacteria lung infection in cystic fibrosis & non-cystic fibrosis patients	Pilot study	Completed	15	07/12/2020	10/10/2022	Australia
Nitric oxide releasing sinus irrigation	NCT04163978	Chronic sinusitis	2	Completed	56	27/20/2019	03/05/2022	Canada
Inhaled nitric oxide	NCT03748992	Pulmonary non-tuberculous mycobacterial infection	2	Completed	10	28/01/2019	26/03/2020	USA

## Microbiome Manipulation

On 30 November 2022, the FDA approved REBYOTA®, the first live biotherapeutic faecal microbiota [205, 206], prepared from human stools donated by screened individuals and administered by enema, for the treatment of individuals ≥18 years following antibacterial treatment for recurrent *Clostridioides difficile* infection (CDI). Subsequently, on 26 April 2023, the first oral therapy of faecal microbiota (VOWST™) was approved by the FDA [207]. CDI is often seen as a complication of antibiotic therapy, resulting in the disruption of the normal gut microbiota and is usually treated with metronidazole or vancomycin. However, with increasing drug-resistant strains, the incidence and even mortality rate of refractory CDI is increasing worldwide. In 10%–60% of cases, the infection returns after completing antibacterial therapy or may not subside at all. For such cases, faecal transplantation may be a considerably more effective option, preventing complications such as, colectomy where mortality rates have risen to 50% following this procedure [208]. Severe illness with CDI can ultimately be fatal, therefore it is essential to choose the correct treatment, and it has been proven that faecal microbiota transplantation (FMT) can provide mortality benefit in critically ill patients, with 77% less mortality rates than with standard antibiotic care [208]. Such microbiome manipulation restores and harmonises the natural gut microbiota, replenishing bacterial balance.

The human gut microbiome generally has a good symbiotic relationship with the host providing (i) a defence against harmful pathogens through competitive exclusion by means of modulating the immune system and producing antimicrobials and (ii) nutritional benefits. The GI tract may also harbour opportunistic colonising pathogenic organisms and under certain conditions such as antibiotic use, acquisition of pathogens during hospitalisation, poor diet/nutrition, physical stress, mental stress, travel, pollution, age and pregnancy can result in a dysbiosis, thereby negatively impacting on the gut microbiota’s mechanisms to prevent the increase in the colonisation of harmful pathogens such as MDR organisms (MDROs), including ESBL producing *Enterobacteriaceae*, carbapenemase-producing *Enterobacterales* (CPE) and vancomycin-resistant enterococci (VRE) as well as contributing to the gut resistome [209].

The precise mechanism of FMT is yet to be determined, however it is speculated that the healthy donor gut flora repopulates the surroundings with normal gut flora with recent research showing that FMT is effective for decolonising [210, 211] and eradicating the carriage of drug and MDR bacteria and antibiotic resistance genes [212]. Such manipulation and modulation of the gut microbiota therefore has a potential role in the therapeutic challenges associated with AMR in terms of treatment and prevention [213].

Research has suggested that decolonisation or eradication of MDROs in the intestine, by means of FMT lowers the risk of infections and cross-contamination. A randomised control trial by Woodworth and colleagues demonstrated that FMT can result in MDRO decolonisation, protection against recurrent infection and a reduction of AMR by means of strain replacement as evidenced by replacement of extended-spectrum β-lactamase ESBL–producing strains with non-ESBL strains [214]. Some small studies and case studies have also reported that FMT has successfully eliminated ESBL colonisation of *K. pneumoniae* and *E. coli* in the gastrointestinal tract of an immunocompromised patient [215], decolonised an allo-HSCT patient with recurrent carbapenem-resistant *Enterobacteriaceae* (CRE) infections [216] and prevented negative outcomes such as mortality associated with MDRO in allogenic hematopoietic cell transplant patients [217]. Nooij et al. suggested that the effect of FMT could prevail for a number of years as they observed three years subsequent to FMT in patients with recurrent *C*. *difficile* infection, patient resistomes were observed which were donor-like [218]. It is interesting to note, however, that although the total load of resistance genes had decreased, patients still possessed higher numbers of various resistance genes compared to the original donor and that resistance plasmids remained unaffected by the transplantation [218]. Of further interest is the report that FMT has been used to eradicate ESBL-producing *K. pneumoniae* in the case of recurrent urinary tract infections, which are often caused by the transfer of faecal material to the urinary tract [219].

A recent systematic review and meta-analysis of case studies/series and two randomised clinical trials concluded that larger sample size randomised clinical trials are warranted using standardised protocols, so that a definitive conclusion can be made on the role of FMT on decolonisation of antibiotic-resistant organisms [220]. Currently, there are several clinical trials ongoing in relation to FMT and the prevention and decolonisation of antibiotic-resistant bacteria and MDROs (Table 5). Although the potential for FMT is to address AMR challenges, it must be noted that there are issues and aspects which need to be considered before FMT can become a robust and routine clinical therapy, including (i) selection of the ideal recipient, (ii) optimal dosage, (iii) route of delivery, (iv) screening of donor stools for MDR organisms to minimise the chances of invasive disease or death, as previously described [221, 222] (v) duration of clinical effect, (vi) the effect of confounding factors based on patient characteristics such as, but not limited to, genetic factors comorbidities, diet and concurrent medications, (vii) safety risks and long term side effects, (viii) public perception, (ix) regulatory issues, (x) balanced clinical and organisational ethical issues and (xi) an evidence-base to provide patients information to reliably make an informed consent due to currently unanswered points as detailed above [223–225].

**TABLE 5:** Ongoing and recently completed clinical trials, within the last 5 years, as of May 2025, relating to faecal microbiota transplantation (FMT) to treat bacterial infections, other than *Clostridioides difficile* or eradicate colonisation or restore gut microbiome.

Clinical Trials.gov ID	Condition	Phase	Status	Enrolment	Start date	Completion/ estimated date	Country
NCT06970262	Effect of FMT on intestinal microbiota and pulmonary microecology in critically ill patients with multidrug-resistant organism infections	N/A	Not yet recruiting	150	15/05/2025	31/12/2026	China
NCT05981430	FMT for decolonization of carbapenem-resistant *Enterobacteriaceae*	N/A	Not yet recruiting	80	01/01/2024	19/08/2025	Hong Kong
NCT06250413	FMT to restore gut microbiome after treatment with antibiotics	N/A	Not yet recruiting	40	02/2024	12/2027	Finland
NCT06641778	FMT in patients with multiple drug resistant *Klebsiella pneumoniae* pneumonia	N/A	Recruiting	100	03/12/2024	31/06/2026	China
NCT05632315	The impact of FMT using the Penn microbiome therapy products on recipient and environmental colonization with multidrug-resistant organisms	2	Recruiting	150	19/08/2024	01/2026	USA
NCT05035342	FMT to eradicate colonizing emergent superbugs	3	Recruiting	214	11/01/2024	04/2028	France
NCT05791396	FMT to eradicate intestinal colonization by carbapenem-resistant *Enterobacteriaceae*	1/2	Recruiting	36	08/02/2024	04/2026	Italy
NCT06156956	FMT to eradicate antibiotic-resistance bacteria from the gastrointestinal tract of patients at high risk of infection and/or to cut off the spread of bacteria with dangerous mechanisms of antibiotic resistance	N/A	Recruiting	200	27/10/2023	31/12/2025	Poland
NCT06461208	FMT to improve the primary outcome (first hospitalisation due to infection) in patients with cirrhosis over 24 months	3	Recruiting	300	21/06/2023	30/11/2026	UK
NCT06050148	FMT as means of preventing recurrent urinary tract infections	2/3	Recruiting	100	08/01/2023	31/12/2029	Finland
NCT06782880	Prevention of infectious complications after liver transplantation	N/A	Recruiting	144	01/05/2023	31/05/2026	Italy
NCT04759001	FMT to eradicate gut colonisation from carbapenem-resistant *Enterobacteriaceae*	1/ 2	Recruiting	52	18/02/2021	15/06/2026	Italy
NCT04583098	FMT on the decolonization of carbapenem-resistant *Enterobacteriaceae* or vancomycin-resistant enterococci in the gut	Observational	Recruiting	100	14/03/2019	31/08/2024	Korea
NCT02543866	FMT as a strategy to eradicate intestinal carriage of resistant organisms	1	Recruiting	20	17/02/2017	09/2026	USA
NCT04014413	Safety and efficacy of FMT: A pilot study	N/A	Recruiting	450	15/07/2019	31/10/2030	Hong Kong
NCT04790565	FMT for eradication of carbapenem-resistant *Enterobacteriaceae* colonization	2/3	Completed	15	01/04/2021	30/06/2022	Israel
NCT03367910	Prevention of recurrent urinary tract infections due to multidrug resistant organisms	1/2	Completed	1	08/02/2018	31/12/2021	USA
NCT04146337	FMT for eradication of carbapenem-resistant *Enterobacteriaceae* colonization	2/3	Completed	3	12/10/2020	30/06/2022	Israel
NCT05461833	FMT in patients with post-infection irritable bowel syndrome	N/A	Completed	59	01/09/2020	15/01/2022	Ukraine
NCT02312986	FMT to reverse multi-drug resistant organism carriage	1	Completed	1	08/2015	31/07/2020	USA
NCT03050515	FMT for the treatment of recurrent urinary tract infections	1	Completed	12	05/02/2018	23/02/2020	USA
NCT03029078	FMT to eradicate digestive tract colonization of patients harbouring extreme drug resistant (XDR) bacteria	4	Completed	50	01/11/2014	01/12/2020	France
NCT04746222	FMT for intestinal carbapenemase-producing *Enterobacteriaceae* decolonization	2/3	Unknown	108	07/2021	07/2023	Singapore

For further information see two recent systematic reviews on FMT which focus on antibiotic-resistant bacteria [226] and CRE [227].

## Predatory Bacteria

Predatory bacteria which have the ability to kill and ingest other bacteria are classified as “living antibiotics” [228–230]. These ubiquitous prokaryotes are found in soils and aquatic environments including seawater, rivers and wastewater and are classified according to their feeding behaviour, falling into two categories: obligate and facultative predators. The predator most studied is *Bdellovibrio bacteriovorus* which is a Gram-negative, δ-proteobacteria and one of the obligatory predatory bacteria classified under the umbrella terminology *Bdellovibrio* and like organisms (BALOs) and its prey are Gram-negative bacteria [228, 231].

*Bdellovibrio bacteriovorus* has been examined for its potential as an antibacterial approach to treat Gram-negative infections and has the characteristics, which in theory correlate with an ideal therapeutic agent in that it (i) can kill Gram-negative organisms in a short time, in less than 30 minutes, and as such would not permit its prey to mount a defence quickly; (ii) does not result in the autolysis of its prey and hence inflammatory molecules are not released and (iii) as prey recognition and attachment is not dependent on a single receptor and following invasion the upregulation of prey destructive enzymes, both in terms of number and diversity, suggests that Gram-negative resistance to *Bdellovibrio bacteriovorus* is unlikely to occur [228, 232].

Various cell and animal models have been utilised to investigate the mechanism of prey recognition and therapeutic potential of these predatory bacteria and Atterbury and Tyson have extensively reviewed such studies in relation to the therapeutic potential of *Bdellovibrio* [228]. More recently, in a rabbit model of endogenous endophthalmitis, it was demonstrated that the *Bdellovibrio bacteriovorus* and *Micavibrio aeruginosavorus* reduced the proliferation of isolates of a fluoroquinolone-resistant *P. aeruginosa* and *Serratia marcescens* [233], highlighting their potential as a novel approach to treat antibiotic-resistant organisms. To date, studies relating to predation susceptibility of clinical isolates are limited, however, an interesting study by Saralegui et al. demonstrated the predation efficiency of *Bdellovibrio bacteriovorus* on clinical isolates of *P*. *aeruginosa* relating to CF and bloodstream infections [234]. The key findings in this study indicated that there was no correlation with predation activity in relation to *P. aeruginosa* genetic lineage, and in the case of CF, isolates of mucoid or non-mucoid phenotype or antibiotic susceptibility phenotype, but there was a higher qualitative and quantitative susceptibility to *Bdellovibrio bacteriovorus* of CF isolates in comparison to bloodstream infection isolates [234]. From the findings of such research, it re-enforces the importance of characterising each predator’s specific prey spectrum if such predators are to be considered as “living antimicrobial agents” to ensure that predation resistance is overcome in relation to specific prey species and individual strains within the species, as the specific prey spectrum of each predator can vary depending on the source of the predator, as well as the source of the prey.

## Immunotherapy

Immunotherapy approaches target the host immune system and have been extensively and primarily used in the treatment of cancers but also autoimmune diseases e.g., multiple sclerosis, rheumatoid arthritis. More recently due to the similarities between cancer and persistent bacterial infections in relation to immune suppression and dysregulation particularly in the case of infectious bacteria which result in granulomas e.g. tuberculosis and non-tuberculous mycobacteria, interest and research has been turned to immunotherapeutic approaches as a potential treatment [235]. McCulloch and colleagues discuss in-depth the mechanisms by which bacteria “hijack” the host immune response resulting in immunosuppression and suggest how therapeutic approaches could manage macrophage function, block immunoregulatory pathways and promote the killing of pathogens in infected cells and contribute to macrophage bactericidal mechanisms [235]. Various immunotherapy approaches have been investigated for infections including some of the WHO priority agents such as *Acinetobacter baumannii*, *P. aeruginosa*, *S*. *aureus*, MRSA, *Streptococcus pneumoniae* and *M*. *tuberculosis,* with the majority of studies in preclinical and some limited clinical trials [235, 236]. Several immunotherapy approaches are currently being investigated including (i) immunomodulators such as checkpoint inhibitors whereby monoclonal antibodies are used to block regulatory molecules (checkpoints) which have reduced the clearance of pathogens and in turn restore the host’s immune function; (ii) cytokine therapy to aid in the regulation of cellular processes including inflammation, humoral immunity and immunosuppression and (iii) cellular therapy using genetically engineered immune cells such as chimeric antigen receptor T cells [235]. (See [236] for a comprehensive list of clinical trials). Immunotherapeutic approaches including the use of vaccines have a realistic potential in the treatment of antibiotic-resistant organisms and offer numerous advantages over conventional antibiotic therapy.

## Vaccine Development to Tackle AMR

The primary goal of vaccination is to protect individuals from becoming ill and to reduce the transmission of pathogens which is of particular importance in the case of infections caused by both antibiotic-resistant and susceptible pathogens. The goal of vaccination is to decrease such infections, resulting in both a decrease in transmission, ideally by herd immunity, the prevention of secondary complications, a reduction in antibiotic use as well as the evolution of antibiotic resistance genes and the development of antibiotic resistance in individuals, thereby preserving effective antibiotics [237, 238].

**FIGURE 5:**
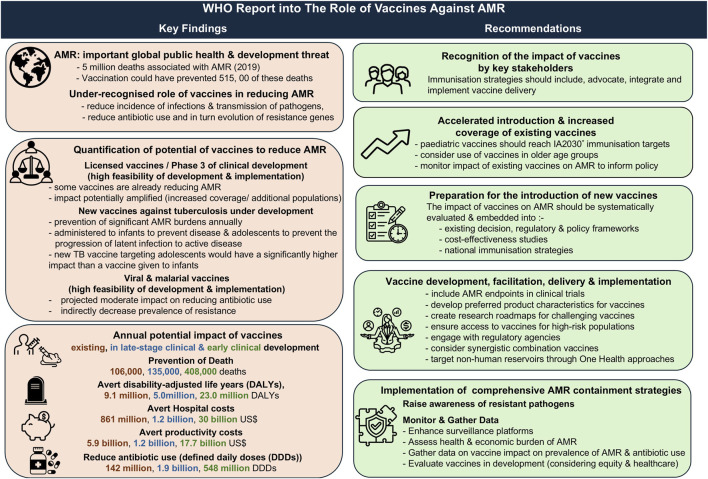
A summary of the key findings and recommendations published by the World Health Organization (WHO) in relation to, “Estimating the impact of vaccines in reducing antimicrobial resistance and antibiotic use”. AMR, antimicrobial resistance; *Immunization Agenda 2030 (https://www.immunizationagenda2030.org/).

Recently, the WHO produced a report which evaluated the potential impact of human vaccines already licensed, in clinical development or hypothesised, in terms of reducing AMR and antibiotic use [238]. This report was a cumulation of a robust evaluation conducted over a two-year period, during which two newly created technical groups relating to vaccines and AMR provided expert knowledge and critical evaluation of data analyses. The purpose of this report was to serve as a guide to the feasibility of vaccines of priority, in relation to biological, product development and implementation feasibility [237, 238] and was intended to promote the recognition of the role vaccines offer in the fight against AMR and as such, the report will be of interest to various global stakeholders, including funding bodies, individuals involved in both vaccine research and development and clinical trials, national decision and policymakers, healthcare workers, civil societies or organisations involved in public health and other non-governmental organisations, as well as regulators. Twenty-four pathogens were selected, based on three criteria namely (i) high incidence of resistance; (ii) resistant pathogens with a high mortality rate and/or (iii) the high volume of antibiotics used in treating such infections [238]. The pathogens included the bacteria, *A*. *baumannii*, *Campylobacter jejuni*, *C*. *difficile*, *Enterococcus faecium*, Enterotoxigenic *Escherichia coli* (ETEC), Extraintestinal pathogenic *Escherichia coli* (ExPEC), Group A *Streptococcus* (GAS), *Haemophilus influenzae* type b (Hib), *Helicobacter pylori*, *K*. *pneumoniae*, *M*. *tuberculosis*, *N*. *gonorrhoeae*, Nontyphoidal *Salmonella*, *P*. *aeruginosa*, *Salmonella* Paratyphi A, *Salmonella* Typhi, *Shigella*, *Staphylococcus aureus* and *Streptococcus pneumoniae*, the parasite *Plasmodium falciparum* as well as four viruses, Influenza, Norovirus, Rotavirus and Respiratory syncytial virus (RSV) [238]. This 168-page report provides an in-depth analysis of how such vaccines could avert deaths, disability-adjusted life years (DALYs), in-hospital costs and daily doses (DDs) annually, all associated with AMR, as well as promoting the importance of enhancing surveillance and awareness of AMR and antibiotic use, particularly when assessing the value of vaccines in development. An infographic of some of the key messages of this report is shown in Figure 5. See section below in relation to the potential role of artificial intelligence (AI) in vaccine development.

## Advances in Technology

There have been several key advances in technology which have enhanced in the search, development and research of novel antimicrobial therapies and their potential efficacy.

### Artificial Intelligence

There has been a recent expansion in the scientific literature of research groups using various artificial intelligence [239], machine learning (ML) formulae and deep learning algorithms to aid in antimicrobial repurposing and drug discovery by mining for novel antibiotic compounds both from virtual libraries [240, 241] and natural sources, such as traditional medicines [242] and predicting potential compounds with antibacterial efficacy against multiple bacteria including carbapenem-resistant *Enterobacteriaceae* and pan-resistant *A*. *baumannii* [240], antibiotic-resistant *B*. *cenocepacia*, ESKAPE pathogens [241], *M*. *tuberculosis* [243] and *M*. *abscessus* [244], as well as predicting toxicity [245]. See [245] for a comprehensive overview of the algorithms and types of learning used in antimicrobial drug discovery and screening and [246] for a glossary of key artificial intelligence (AI) terms.

AI has been used to mine biological sequences contained within large databases for AMPs as well as to generate potential AMPs and predict the properties, activity and toxicity of such AMPs [75, 239]. Such technological approaches which encompass prediction models, not only expedite the discovery of antimicrobials but may also lead to the discovery of new classes of antimicrobial drugs which can be subsequently validated by *in vitro*, *in vivo* studies and clinical trials. AI has also been used in the development of other small molecule antibacterial drugs, development of bacteriophage therapy from identification, prediction of phage virion proteins, host prediction and interactions and lifestyle prediction and the discovery of antibacterial essential oils [247]. Two recent review articles have detailed antibacterial drugs which were developed using AI, which target antibiotic-resistant and MDR Gram-negative and Gram-positive organisms including, Carbapenem-resistant *Acinetobacter baumannii* (CRAB), Carbapenem-Resistant *Pseudomonas aeruginosa* (CRPA), carbapenem-resistant *Klebsiella pneumoniae* (CRKP), Carbapenem-resistant *Enterobacterales* (CRE), MDR *E. coli*, as well as priority pathogens *P. aeruginosa, M. tuberculosis and A. baumannii*, amongst others [247, 248].

AI and deep learning in conjunction with convolutional neural networks (CNN), recurrent neural networks (RNN) and data mining, can also be used to identify disease patterns and aid in the detection and identification of pathogens, the diagnosis of infection, the prediction of AMR development and determination of antimicrobial susceptibility which can aid in the development and guidance of clinical decisions including therapeutic treatment [239, 249]. On a cautionary note, the use of AI technologies utilising machine learning or large language models to offer guidance on antimicrobial therapy have their limitations and several concerns as detailed in a recent systematic review, particularly in relation to prescribing errors, safety risks and the management of complex clinical cases. It is therefore essential that input and final clinical decisions are made by infectious disease clinicians [250].

AI and ML based approaches have been used to aid in the understanding of infectious diseases as well as in the selection of components of vaccines and the identification of potential antigens which could trigger appropriate effective host immune responses, with a long duration and efficacy [251]. The use of high throughput genomic and proteomic data can be used to build predictive models of infection and vaccine design, thereby reducing the time historically required to detect relevant antigens. Approaches such as reverse vaccinology which uses bacterial genomes and computational informatics led to the successful development of the Bexsero 4CMenB meningococcal vaccine which is now routinely used in the UK child immunisation schedule [252]. Kaushik et al. critically evaluated the use of AI-based approaches, including AI-powered reverse vaccinology platforms to aid in the development of vaccines and summarised research which has been conducted to date on MDR bacteria such as *P. aeruginosa, S. pneumoniae, K. pneumoniae, E. coli, S. aureus* and *A. baumannii*. Although the value of AI in vaccinology is recognised, there are practical concerns and limitations which should be acknowledged including the difficulty associated with intensive computation and the requirement of skilled personnel to implement and interpret such data, the availability of limited data sets and the reliability on commercial tools. It is also important that AI complements rather than replaces conventional laboratory vaccinology approaches which are required to determine vaccine efficacy and safety. Of note, in September 2025, The University of Oxford in partnership with the Ellison Institute of Technology, received research funding of £118m to use AI to help elucidate the immunogenic mechanisms of pathogens such as *S. pneumoniae, S. aureus* and *E. coli*, amongst others, to help in the development of vaccines which are urgently needed [253]. This highlights that AI will continue to have a prominent role in the development of vaccines against MDR bacteria of major clinical concern.

### Organ-on-a-Chip

A novel technology, organ-on-a-chip (OOC), is an *in vitro* 3D microfluidic model which is superior to conventional 2-D and 3D- cell culture models and animal models. It can be used for the purposes of infectious disease modelling [254], drug discovery/screening [255], the preclinical evaluation of drug target sites, drug absorption, distribution, metabolism, and excretion (ADME) and drug toxicity [256, 257]. OOCs offer *in vitro* models which allow cells to be maintained in an environment which is more representative of the *in vivo* human physiology, with microchannels enabling the control of the cellular environment and facilitation of the examination in relation to both biochemical and physical cues [257]. OOC models mimic the pathophysiology of a specific organ or tissue and they can be used in conjunction with multiple OOCs (MOOCs) to mimic the multi-organ systems in the human body, e.g. a MOOC, comprising of skin, liver, kidney and intestine to evaluate ADME [257, 258]. Kidney OOCs have been used to examine antibiotic nephrotoxicity induced by ciprofloxacin, gentamicin and a novel formulation of polymyxin B [259, 260].

The superiority of this technology offers the benefit of decreasing the use of animal models, as they can be used to screen for toxicity of novel drugs prior to pre-clinical trials, which aligns with the recent 2022 change to the FDA legislation, regarding new medicines in that potential drugs no longer need to be tested for safety and efficacy in animals but the use of other non-animal models prior to human trials is permitted [261]. This change has been welcomed by animal welfare organisations as well as OOC researchers, particularly as more than nine in ten drugs which undergo clinical trials ultimately fail as they are unsafe or ineffective [261].

It must also be acknowledged that there are also a number of limitations in relation to (i) the lack of standardisation including, but not limited to, defining OOC, the manufacturing of OOC, including materials used and the production process, sources of cells and biological materials, sterility, quality and test protocols [262], (ii) the development of a universal medium when using different cell types and organs in the case of MOOC, (iii) technical issues such as the absorption of drugs and small molecules (<1 kDa) to polymeric materials such as polydimethylsiloxane (PDMS), which are used in many OOC devices, (iv) challenges in replicating the interactions between various organs in the host, (v) the lack of current regulation and (vi) cost effectiveness [263]. Steps have been taken in relation to addressing these issues, particularly relating to standardisation by the development of a roadmap for OOC standardisation published by the European Commission’s Joint Research Centre (JRC) [264] and The UK Organ-on-a-Chip Technologies Network [265].

The OOC technology is currently being developed in relation to vaccination research by various groups [266] including the UK Health Security Agency’s Vaccine Development and Evaluation Centre (VDEC) [267]. Such advances have included the development of MOOC chip models including an OOC lymph node [268]. As such, the future of this OOC approach could prove beneficial to the development and evaluation of vaccines targeting pathogens, including antibiotic-resistant organisms. Furthermore, with OOC models using host stem cells could facilitate personalised medicine [269] and particularly in relation to antibacterial therapy and prevention. A similar microfluidic technology, known as lab-on-a-chip has recently been proposed to simplify and minimise the time taken to predict antimicrobial susceptibility, which has the potential to enhance in the selection of appropriate and efficacious therapeutic options [270].

## Conclusion

In conclusion, the ever-increasing development of antibiotic resistance is causing global problems in terms of treatment of infectious diseases and novel antimicrobials and therapeutic approaches have been researched and developed in an attempt to decrease the mortality and morbidity associated with AMR. This overview of current research of novel approaches to prevent and treat infections particularly those which are MDR and associated WHO priority pathogens, highlights the global efforts which research groups continue to make in an attempt to diminish the impact of AMR on human health. Such innovations offer a welcome prospect, however, considerable challenges remain in terms of regulatory development, delivery, efficacy, safety and clinical evaluation and application. Although the search for novel antimicrobials continues and many of these therapeutic approaches have demonstrated proof of concept, it must be recognised that these are still in pre-clinical or in early stages of clinical trials. It is therefore essential that funding remains available for further development and more clinical trials which are required to fully evaluate the safe clinical applicability of such novel molecules and approaches. Furthermore, to date, research has focused on a narrow spectrum of pathogens and specific patient groups, which would require broader expansion due to the rise of antibiotic-resistant organisms causing infection both in the community and healthcare settings. Moving forward, the complexity of AMR as well as the growing number of innovations to combat AMR as discussed in this review, present challenges as to how best to proceed at local, national and international level. Various governments have attempted to integrate these factors by producing strategic plans, such as the “UK 5-year action plan for antimicrobial resistance 2024 to 2029” [271] and the EU’s “Council Recommendation on stepping up EU actions to combat antimicrobial resistance in a One Health approach” [272]. The future is optimistic in that these novel approaches, although not replacements for conventional antibiotic therapy, could have a synergistic and/or reversal role alongside conventional antimicrobial therapy, to provide a realistic mechanism to overcome the challenges associated with AMR.

## References

[1] O’Neill J . Review on Antimicrobial Resistance. Antimicrobial Resistance: Tackling Drug-Resistant Infections Globally: Final Report and Recommendations (2016). Available online at: https://amr-review.org/ (Accessed July 01, 2025).

[2] Tang KWK Millar BC Moore JE . Antimicrobial Resistance (AMR). Br J Biomed Sci (2023) 80:11387. 10.3389/bjbs.2023.11387 37448857 PMC10336207

[3] World Health Organization. WHO Bacterial Priority Pathogens List, 2024: Bacterial Pathogens of Public Health Importance to Guide Research, Development and Strategies to Prevent and Control Antimicrobial Resistance (2024). Available online at: https://www.who.int/publications/i/item/9789240093461 (Accessed July 01, 2025).

[4] World Health Organization. Monitoring and Evaluation of the Global Action Plan on Antimicrobial Resistance (2019). Available online at: https://www.who.int/publications/i/item/monitoring-and-evaluation-of-the-global-action-plan-on-antimicrobial-resistance (Accessed July 01, 2025).

[5] Mancuso G Midiri A De Gaetano S Ponzo E Biondo C . Tackling Drug-Resistant Tuberculosis: New Challenges from the Old Pathogen *Mycobacterium tuberculosis* . Microorganisms (2023) 11(9):2277. 10.3390/microorganisms11092277 37764122 PMC10537529

[6] World Health Organization. WHO Announces Updated Definitions of Extensively Drug-Resistant Tuberculosis (2021). Available online at: https://www.who.int/news/item/27-01-2021-who-announces-updated-definitions-of-extensively-drug-resistant-tuberculosis (Accessed July 01, 2025).

[7] Golparian D Cole MJ Sánchez-Busó L Day M Jacobsson S Uthayakumaran T Antimicrobial-Resistant Neisseria gonorrhoeae in Europe in 2020 Compared with in 2013 and 2018: A Retrospective Genomic Surveillance Study. Lancet Microbe (2024) 5(5):e478–e488. 10.1016/S2666-5247(23)00370-1 38614111

[8] Perikleous EP Gkentzi D Bertzouanis A Paraskakis E Sovtic A Fouzas S . Antibiotic Resistance in Patients with Cystic Fibrosis: Past, Present, and Future. Antibiotics (Basel) (2023) 12(2):217. 10.3390/antibiotics12020217 36830128 PMC9951886

[9] Nanayakkara AK Boucher HW Fowler VG Jr Jezek A Outterson K Greenberg DE . Antibiotic Resistance in the Patient with Cancer: Escalating Challenges and Paths Forward. CA Cancer J Clin (2021) 71(6):488–504. 10.3322/caac.21697 34546590

[10] Halawa EM Fadel M Al-Rabia MW Behairy A Nouh NA Abdo M Antibiotic Action and Resistance: Updated Review of Mechanisms, Spread, Influencing Factors, and Alternative Approaches for Combating Resistance. Front Pharmacol (2024) 14:1305294. 10.3389/fphar.2023.1305294 38283841 PMC10820715

[11] Schroeder M Brooks BD Brooks AE . The Complex Relationship Between Virulence and Antibiotic Resistance. Genes (Basel) (2017) 8(1):39. 10.3390/genes8010039 28106797 PMC5295033

[12] García-Castro M Sarabia F Díaz-Morilla A López Romero JM . Approved Antibacterial Drugs in the Last 10 Years: From the Bench to the Clinic. Explor Drug Sci (2023) 1:180–209. 10.37349/eds.2023.00013

[13] Shi Z Zhang J Tian L Xin L Liang C Ren X A Comprehensive Overview of the Antibiotics Approved in the Last Two Decades: Retrospects and Prospects. Molecules (2023) 28(4):1762. 10.3390/molecules28041762 36838752 PMC9962477

[14] Zasheva A Batcheva E Ivanova KD Yanakieva A . Differences in Patient Access to Newly Approved Antibacterial Drugs in EU/EEA Countries. Antibiotics (Basel) (2024) 13(11):1077. 10.3390/antibiotics13111077 39596770 PMC11591277

[15] Theuretzbacher U . Evaluating the Innovative Potential of the Global Antibacterial Pipeline. Clin Microbiol Infect (2025) 31(6):903–9. 10.1016/j.cmi.2023.09.024 37805036

[16] Veve MP Wagner JL . Lefamulin: Review of a Promising Novel Pleuromutilin Antibiotic. Pharmacotherapy (2018) 38(9):935–46. 10.1002/phar.2166 30019769

[17] Poultry DVM . Tiamulin (2025). Available online at: https://www.poultrydvm.com/drugs/tiamulin (Accessed July 01, 2025).

[18] European Medicines Agency. Xenlata (2020). Available online at: https://www.ema.europa.eu/en/medicines/human/EPAR/xenleta#product-info (Accessed July 01, 2025).

[19] Messenger AM Barnes AN Gray GC . Reverse Zoonotic Disease Transmission (Zooanthroponosis): A Systematic Review of Seldom-Documented Human Biological Threats to Animals. PLoS One (2014) 9(2):e89055. 10.1371/journal.pone.0089055 24586500 PMC3938448

[20] Watkins RR Thapaliya D Lemonovich TL Bonomo RA . Gepotidacin: A Novel, Oral, 'First-In-Class' Triazaacenaphthylene Antibiotic for the Treatment of Uncomplicated Urinary Tract Infections and Urogenital Gonorrhoea. J Antimicrob Chemother (2023) 78(5):1137–42. 10.1093/jac/dkad060 36883591

[21] Ross JDC Wilson J Workowski KA Taylor SN Lewis DA Gatsi S Oral Gepotidacin for the Treatment of Uncomplicated Urogenital Gonorrhoea (EAGLE-1): A Phase 3 Randomised, Open-Label, Non-Inferiority, Multicentre Study. Lancet (2025) 405(10489):1608–20. 10.1016/S0140-6736(25)00628-2 40245902

[22] US Government. Public Law 112–144 112th Congress. Food Drug Adm Saf Innovation Act (2012). Available online at: https://www.congress.gov/112/plaws/publ144/PLAW-112publ144.pdf (Accessed July 01, 2025).

[23] Food and Drug Administration. Food and Drug Administration Safety and Innovation Act (FDASIA) (2018). Available online at: https://www.fda.gov/regulatory-information/selected-amendments-fdc-act/food-and-drug-administration-safety-and-innovation-act-fdasia (Accessed July 01, 2025).

[24] Food and Drug Administration. Qualified Infectious Disease Product Designation-Questions and Answers Guidance for Industry (2021). Available online at: https://www.fda.gov/media/148480/download (Accessed July 01, 2025).

[25] Hoy SM . Contezolid: First Approval. Drugs (2021) 81(13):1587–91. 10.1007/s40265-021-01576-0 34365606 PMC8536612

[26] MicuRx. Micurx Receives FDA Qualified Infectious Disease Product (QIDP) and Fast Track Designation for Contezolid and Contezolid Acefosamil (2023). Available online at: https://www.micurx.com/1641.html (Accessed July 01, 2025).

[27] Jiang G Liu R Xue Y Ge Q Nie L Lv Z Contezolid Harbored Equivalent Efficacy to Linezolid in Tuberculosis Treatment in a Prospective and Randomized Early Bactericidal Activity Study. Infect Drug Resist (2025) 18:261–8. 10.2147/IDR.S499816 39830036 PMC11742132

[28] Chen Y Ren J Li F Ye X Wu Y . The Antibiotic Therapy Containing Contezolid Successfully Treated methicillin-Sensitive *Staphylococcus aureus* Infective Endocarditis Accompanied with Cerebrovascular Complications. BMC Infect Dis (2024) 24(1):1301. 10.1186/s12879-024-10157-x 39543478 PMC11566642

[29] Zhou S Xin C Liu W . Sequential Therapy of Linezolid and Contezolid to Treat Hematogenous Lung Abscess Caused by *Staphylococcus aureus* in a Congenital Cerebral Hypoplasia Patient: A Case Report. Infect Drug Resist (2025) 18:253–60. 10.2147/IDR.S502839 39830034 PMC11740585

[30] Guo Q Xu L Tan F Zhang Y Fan J Wang X A Novel Oxazolidinone, Contezolid (MRX-I), Expresses Anti-*Mycobacterium Abscessus* Activity in Vitro. Antimicrob Agents Chemother (2021) 65(11):e0088921. 10.1128/AAC.00889-21 34460305 PMC8522767

[31] Chen P An L Zhang Z . Sequential Therapy of Linezolid and Contezolid to Treat Vancomycin-Resistant *Enterococcus faecium* Pneumonia in a Centenarian Patient: Case Report. Infect Drug Resist (2023) 16:1573–8. 10.2147/IDR.S401533 36969942 PMC10032165

[32] Zhang X Huang H Wang J Wei B . Efficacy of Contezolid in the Treatment of Catheter-Related Bloodstream Infections Caused by Methicillin-Resistant *Staphylococcus aureus* in a Patient with Hepatorenal Syndrome and Acute Kidney Injury: A Case Report. Infect Drug Resist (2025) 18:307–11. 10.2147/IDR.S501604 39835163 PMC11745060

[33] Dong J Cheng Q Tang C Zhong Y Wang J Lv M Comparative in Vitro Drug Susceptibility Study of Five Oxazolidinones Against *Mycobacterium tuberculosis* in Hainan, China. Pathogens (2025) 14(3):218. 10.3390/pathogens14030218 40137704 PMC11945096

[34] Yang W Li X Chen J Zhang G Li J Zhang J Multicentre Evaluation of *In Vitro* Activity of Contezolid Against Drug-Resistant *Staphylococcus* and *Enterococcus* . J Antimicrob Chemother (2024) 79(12):3132–41. 10.1093/jac/dkae331 39315881

[35] Gao X Ding C Xie D Wang Q Jiang P Wang Y Contezolid-Containing Regimen Successfully Treated Multiple Drug Resistance *Mycobacterium abscessus* Complex Infection of Skin: A Case Report and Literature Review. Infect Drug Resist (2024) 17:1243–9. 10.2147/IDR.S453541 38560704 PMC10981868

[36] An H Sun W Liu X Wang T Qiao J Liang J . *In vitro* Activities of Contezolid (MRX-I) Against Drug-Sensitive and Drug-Resistant *Mycobacterium tuberculosis* . Microbiol Spectr (2023) 11(5):e0462722. 10.1128/spectrum.04627-22 37732805 PMC10580816

[37] Huang Y Chiaraviglio L Bode-Sojobi I Kirby JE . Triple Antimicrobial Combinations with Potent Synergistic Activity Against *M. abscessus* . Antimicrob Agents Chemother (2025) 69(4):e0182824. 10.1128/aac.01828-24 40084880 PMC11963555

[38] Matlock A Garcia JA Moussavi K Long B Liang SY . Advances in Novel Antibiotics to Treat Multidrug-Resistant Gram-Negative Bacterial Infections. Intern Emerg Med (2021) 16(8):2231–41. 10.1007/s11739-021-02749-1 33956311 PMC8100742

[39] Sargianou M Stathopoulos P Vrysis C Tzvetanova ID Falagas ME . New β-Lactam/β-Lactamase Inhibitor Combination Antibiotics. Pathogens (2025) 14(4):307. 10.3390/pathogens14040307 40333039 PMC12029989

[40] Pfizer. EMBLAVEO® (aztreonam/Avibactam) (2025). Available online at: https://www.pfizerpro.co.uk/medicine/emblaveo (Accessed July 01, 2025).

[41] Keam SJ . Sulbactam/Durlobactam: First Approval. Drugs (2023) 83(13):1245–52. 10.1007/s40265-023-01920-6 37523122

[42] Medicines and Healthcare products Regulatory Agency. Combined Antibiotic Approved to Treat Adult Patients with Severe Infections of the Urinary Tract and Hospital-Acquired Pneumonia (2024). Available online at: https://www.gov.uk/government/news/combined-antibiotic-approved-to-treat-adult-patients-with-severe-infections-of-the-urinary-tract-and-hospital-acquired-pneumonia (Accessed July 01, 2025).

[43] Bhowmick T Canton R Pea F Quevedo J Santerre Henriksen A Timsit JF Cefepime-Enmetazobactam: First Approved Cefepime-β- Lactamase Inhibitor Combination for Multi-Drug Resistant Enterobacterales. Future Microbiol (2025) 20(4):277–86. 10.1080/17460913.2025.2468112 40007489 PMC11938971

[44] Mansour H Ouweini AEL Chahine EB Karaoui LR . Imipenem/Cilastatin/Relebactam: A New Carbapenem β-Lactamase Inhibitor Combination. Am J Health Syst Pharm (2021) 78(8):674–83. 10.1093/ajhp/zxab012 33580649

[45] Duda-Madej A Viscardi S Topola E . Meropenem/Vaborbactam: Β-Lactam/Β-Lactamase Inhibitor Combination, the Future in Eradicating Multidrug Resistance. Antibiotics (Basel) (2023) 12(11):1612. 10.3390/antibiotics12111612 37998814 PMC10668789

[46] Martin-Loeches I Bruno CJ DeRyke CA . Perspectives on the Use of ceftolozane/tazobactam: A Review of Clinical Trial Data and Real-World Evidence. Future Microbiol (2024) 19(6):465–80. 10.2217/fmb-2023-0197 38252038 PMC11216532

[47] Wistrand-Yuen P Olsson A Skarp KP Friberg LE Nielsen EI Lagerbäck P Evaluation of Polymyxin B in Combination with 13 Other Antibiotics Against Carbapenemase-Producing *Klebsiella pneumoniae* in Time-Lapse Microscopy and Time-Kill Experiments. Clin Microbiol Infect (2020) 26(9):1214–21. 10.1016/j.cmi.2020.03.007 32224200

[48] Cassir N Coiffard B Hadjadj L Bermudez J Okdah L Ailhaud L Multi-Target Combination of Antibiotics as Salvage Therapy for Severe Infection Caused by Pan-*Resistant Burkholderia cenocepacia* Following Lung Transplantation. Transplant Rep (2025) 10(1):100170. 10.1016/j.tpr.2024.100170

[49] Aggarwal M Patra A Awasthi I George A Gagneja S Gupta V Drug Repurposing Against Antibiotic Resistant Bacterial Pathogens. Eur J Med Chem (2024) 279:116833. 10.1016/j.ejmech.2024.116833 39243454

[50] Iskandar IW Nurhasanah A Hatta M Hamid F Handayani I Chaera U Computational Drug Repurposing for Tuberculosis by Inhibiting Ag85 Complex Proteins. Narra J (2025) 5(1):e1130. 10.52225/narra.v5i1.1130 40352212 PMC12059857

[51] Pompilio A Lupetti V Puca V Di Bonaventura G . Repurposing High-Throughput Screening Reveals Unconventional Drugs with Antimicrobial and Antibiofilm Potential Against Methicillin-Resistant *Staphylococcus aureus* from a Cystic Fibrosis Patient. Antibiotics (Basel) (2025) 14(4):402. 10.3390/antibiotics14040402 40298549 PMC12024424

[52] Mohammed MA Ahmed MT Anwer BE Aboshanab KM Aboulwafa MM . Propranolol, Chlorpromazine and Diclofenac Restore Susceptibility of Extensively Drug-Resistant (XDR)-*Acinetobacter Baumannii* to Fluoroquinolones. PLoS One (2020) 15(8):e0238195. 10.1371/journal.pone.0238195 32845920 PMC7449414

[53] Sharma P Kalra A Tripathi AD Chaturvedi VK Chouhan B . Antimicrobial Proficiency of Amlodipine: Investigating Its Impact on *Pseudomonas* spp. in Urinary Tract Infections. Indian J Microbiol (2025) 65(1):347–58. 10.1007/s12088-024-01280-z 40371041 PMC12069773

[54] Raqib R Sarker P . Repurposed Drugs and Plant-Derived Natural Products as Potential Host-Directed Therapeutic Candidates for Tuberculosis. Biomolecules (2024) 14(12):1497. 10.3390/biom14121497 39766204 PMC11673177

[55] Chadha J Khullar L Gulati P Chhibber S Harjai K . Anti-Virulence Prospects of Metformin Against *Pseudomonas Aeruginosa*: A New Dimension to a Multifaceted Drug. Microb Pathog (2023) 183:106281. 10.1016/j.micpath.2023.106281 37541553

[56] Oh J Choi S Seo H Kim DH Kim H Lee D VLX600, an Anticancer Iron Chelator, Exerts Antimicrobial Effects on *Mycobacterium abscessus* Infections. Microbiol Spectr (2025) 20:e0071925. 10.1128/spectrum.00719-25 40539807 PMC12323642

[57] Baraka K Abozahra R Khalaf E Bennaya ME Abdelhamid SM . Repurposing of Paroxetine and Fluoxetine for Their Antibacterial Effects Against Clinical *Pseudomonas aeruginosa* Isolates in Egypt. AIMS Microbiol (2025) 11(1):126–49. 10.3934/microbiol.2025007 40161243 PMC11950684

[58] Di Bonaventura G Lupetti V De Fabritiis S Piccirilli A Porreca A Di Nicola M Giving Drugs a Second Chance: Antibacterial and Antibiofilm Effects of Ciclopirox and Ribavirin Against Cystic Fibrosis *Pseudomonas aeruginosa* Strains. Int J Mol Sci (2022) 23(9):5029. 10.3390/ijms23095029 35563420 PMC9102761

[59] Chadha J Khullar L Gulati P Chhibber S Harjai K . Repurposing Albendazole as a Potent Inhibitor of Quorum Sensing-Regulated Virulence Factors in *Pseudomonas Aeruginosa*: Novel Prospects of a Classical Drug. Microb Pathog (2024) 186:106468. 10.1016/j.micpath.2023.106468 38036112

[60] Zhao L Zhang H Zha L Zhou X Yang M . Bactericidal and Anti-Biofilm Activity of Ebastine Against *Staphylococcus aureus* . Lett Appl Microbiol (2025) 12:ovaf086. 10.1093/lambio/ovaf086 40504561

[61] Chadha J Mudgil U Khullar L Ahuja P Harjai K . Revitalizing Common Drugs for Antibacterial, Quorum Quenching, and Antivirulence Potential Against *Pseudomonas aeruginosa*: *In Vitro* and in Silico Insights. 3 Biotech (2024) 14(10):219. 10.1007/s13205-024-04070-y 39239248 PMC11371971

[62] Cao D Liu G Wang Y Xia X . Repurposing Astemizole to Kill Multidrug-Resistant Bacteria Isolated in General Surgery. Microb Pathog (2025) 200:107369. 10.1016/j.micpath.2025.107369 39929397

[63] Chatupheeraphat C Kaewsai N Anuwongcharoen N Phanus-Umporn C Pornsuwan S Eiamphungporn W . Penfluridol Synergizes with Colistin to Reverse Colistin Resistance in Gram-Negative Bacilli. Sci Rep (2025) 15(1):16114. 10.1038/s41598-025-01303-9 40341530 PMC12062240

[64] Colquhoun JM Brzezinski CU Ji A Marotta J Elsen FAV Bonomo RA Repurposing a Drug to Punish Carbapenem-Resistant *Acinetobacter Baumannii* . Proc Natl Acad Sci U S A (2025) 122(24):e2423650122. 10.1073/pnas.2423650122 40493197 PMC12184509

[65] Shang Y Huang Y Meng Q Yu Z Wen Z Yu F . Fingolimod as a Potent Anti-Staphylococcus Aureus: Ph-Dependent Cell Envelope Damage and Eradication of biofilms/persisters. BMC Microbiol (2025) 25(1):299. 10.1186/s12866-025-03973-x 40380090 PMC12083125

[66] Zhang N Li X Liu X Cheng P Li L Chai Y Aspirin Enhances the Antibacterial Activity of Colistin Against Multidrug-Resistant *Pseudomonas aeruginosa* . Eur J Pharmacol (2025) 997:177480. 10.1016/j.ejphar.2025.177480 40057155

[67] Oliveira IM Borges A Borges F Simões M . Repurposing Ibuprofen to Control *Staphylococcus aureus* Biofilms. Eur J Med Chem (2019) 166:197–205. 10.1016/j.ejmech.2019.01.046 30711830

[68] Bisaro F Jackson-Litteken CD McGuffey JC Hooppaw AJ Bodrog S Jebeli L Diclofenac Sensitizes Multi-Drug Resistant *Acinetobacter baumannii* to Colistin. Plos Pathog (2024) 20(11):e1012705. 10.1371/journal.ppat.1012705 39571043 PMC11620633

[69] Zhu J She P Fu J Peng C Wu Y . Identification of Eltrombopag as a Repurposing Drug Against *Staphylococcus epidermidis* and Its Biofilms. Curr Microbiol (2021) 78(4):1159–67. 10.1007/s00284-021-02386-z 33611618

[70] She P Li S Zhou L Liu Y Xu L Hussain Z Repurposing Eltrombopag as an Antimicrobial Agent Against Methicillin-Resistant *Staphylococcus aureus* . Front Microbiol (2022) 12:790686. 10.3389/fmicb.2021.790686 35140693 PMC8819062

[71] Zhang W Ran J Shang L Zhang L Wang M Fei C Niclosamide as a Repurposing Drug Against Gram-Positive Bacterial Infections. J Antimicrob Chemother (2022) 77(12):3312–20. 10.1093/jac/dkac319 36173387

[72] Lopes BS Hanafiah A Nachimuthu R Muthupandian S Md Nesran ZN Patil S . The Role of Antimicrobial Peptides as Antimicrobial and Antibiofilm Agents in Tackling the Silent Pandemic of Antimicrobial Resistance. Molecules (2022) 27(9):2995. 10.3390/molecules27092995 35566343 PMC9105241

[73] Duarte-Mata DI Salinas-Carmona MC . Antimicrobial Peptides´ Immune Modulation Role in Intracellular Bacterial Infection. Front Immunol (2023) 14:1119574. 10.3389/fimmu.2023.1119574 37056758 PMC10086130

[74] Lin C Xiong S Cui F Zhang Z Shi H Wei L . Deep Learning in Antimicrobial Peptide Prediction. J Chem Inf Model (2025) 8:7373–92. 10.1021/acs.jcim.5c00530 40626654

[75] Szymczak P Zarzecki W Wang J Duan Y Wang J Coelho LP AI-Driven Antimicrobial Peptide Discovery: Mining and Generation. Acc Chem Res (2025) 58(12):1831–46. 10.1021/acs.accounts.0c00594 40459283 PMC12177927

[76] Chen CH Lu TK . Development and Challenges of Antimicrobial Peptides for Therapeutic Applications. Antibiotics (Basel) (2020) 9(1):24. 10.3390/antibiotics9010024 31941022 PMC7168295

[77] Taylor P . Roche Takes New Antibiotic into Phase 3 for 'Urgent Threat (2025). Available online at: https://pharmaphorum.com/news/roche-takes-new-antibiotic-phase-3-urgent-threat (Accessed July 12, 2025).

[78] Zampaloni C Mattei P Bleicher K Winther L Thäte C Bucher C A Novel Antibiotic Class Targeting the Lipopolysaccharide Transporter. Nature (2024) 625(7995):566–71. 10.1038/s41586-023-06873-0 38172634 PMC10794144

[79] Liu HY Prentice EL Webber MA . Mechanisms of Antimicrobial Resistance in Biofilms. NPJ *Antimicrob Resist* . NPJ Antimicrob Resist (2024) 2(1):27. 10.1038/s44259-024-00046-3 39364333 PMC11445061

[80] Rodríguez-Urretavizcaya B Vilaplana L Marco MP . Strategies for Quorum Sensing Inhibition as a Tool for Controlling *Pseudomonas aeruginosa* Infections. Int J Antimicrob Agents (2024) 64(5):107323. 10.1016/j.ijantimicag.2024.107323 39242051

[81] Kaushik V Tiwari M Joshi R Tiwari V . Therapeutic Strategies Against Potential Antibiofilm Targets of Multidrug-Resistant *Acinetobacter baumannii* . J Cell Physiol (2022) 237(4):2045–63. 10.1002/jcp.30683 35083758

[82] Ghosh C Sarkar P Issa R Haldar J . Alternatives to Conventional Antibiotics in the Era of Antimicrobial Resistance. Trends Microbiol (2019) 27(4):323–38. 10.1016/j.tim.2018.12.010 30683453

[83] Howard JJ Sturge CR Moustafa DA Daly SM Marshall-Batty KR Felder CF Inhibition of *Pseudomonas aeruginosa* by Peptide-Conjugated Phosphorodiamidate Morpholino Oligomers. Antimicrob Agents Chemother (2017) 61(4):e01938–16. 10.1128/AAC.01938-16 28137807 PMC5365667

[84] Geller BL Li L Martinez F Sully E Sturge CR Daly SM Morpholino Oligomers Tested *In Vitro*, in Biofilm and *In Vivo* Against Multidrug-Resistant *Klebsiella pneumoniae* . J Antimicrob Chemother (2018) 73(6):1611–9. 10.1093/jac/dky058 29506074 PMC6251509

[85] Meng J Wang H Hou Z Chen T Fu J Ma X Novel Anion Liposome-Encapsulated Antisense Oligonucleotide Restores Susceptibility of Methicillin-Resistant *Staphylococcus aureus* and Rescues Mice from Lethal Sepsis by Targeting *mecA* . Antimicrob Agents Chemother (2009) 53(7):2871–8. 10.1128/AAC.01542-08 19433567 PMC2704696

[86] Sully EK Geller BL Li L Moody CM Bailey SM Moore AL Peptide-Conjugated Phosphorodiamidate Morpholino Oligomer (PPMO) Restores Carbapenem Susceptibility to NDM-1-Positive Pathogens *In Vitro* and *In Vivo* . J Antimicrob Chemother (2017) 72(3):782–90. 10.1093/jac/dkw476 27999041 PMC5890718

[87] Sturge CR Felder-Scott CF Pifer R Pybus C Jain R Geller BL AcrAB-TolC Inhibition by Peptide-Conjugated Phosphorodiamidate Morpholino Oligomers Restores Antibiotic Activity *in Vitro* and *in Vivo* . ACS Infect Dis (2019) 5(8):1446–55. 10.1021/acsinfecdis.9b00123 31119935

[88] Moustafa DA Wu AW Zamora D Daly SM Sturge CR Pybus C Peptide-Conjugated Phosphorodiamidate Morpholino Oligomers Retain Activity Against Multidrug-Resistant *Pseudomonas aeruginosa in Vitro* and *in Vivo. mBio* (2021) 12(1):e02411-20. 10.1128/mBio.02411-20 PMC7844538 33436433

[89] Nanayakkara AK Moustafa DA Pifer R Goldberg JB Greenberg DE . Sequence Specificity Defines the Effectiveness of PPMOs Targeting *Pseudomonas aeruginosa* . Antimicrob Agents Chemother (2023) 67(9):e0024523. 10.1128/aac.00245-23 37610213 PMC10508178

[90] Wang L Hu C Shao L . The Antimicrobial Activity of Nanoparticles: Present Situation and Prospects for the Future. Int J Nanomedicine (2017) 12:1227–49. 10.2147/IJN.S121956 28243086 PMC5317269

[91] Bahrami A Delshadi R Jafari SM . Active Delivery of Antimicrobial Nanoparticles into Microbial Cells Through Surface Functionalization Strategies. Trends Food Sci and Technology (2020) 99:217–28. 10.1016/j.tifs.2020.03.008

[92] Ogunsona EO Muthuraj R Ojogbo E Valerio O Mekonnen TH . Engineered Nanomaterials for Antimicrobial Applications: A Review. Appl Mater Today (2020) 18:100473. 10.1016/j.apmt.2019.100473

[93] Wyszogrodzka G Marszałek B Gil B Dorożyński P . Metal-Organic Frameworks: Mechanisms of Antibacterial Action and Potential Applications. Drug Discov Today (2016) 21(6):1009–18. 10.1016/j.drudis.2016.04.009 27091434

[94] Ribeiro AI Dias AM Zille A . Synergistic Effects Between Metal Nanoparticles and Commercial Antimicrobial Agents: A Review. ACS Appl Nano Mater (2022) 5(3):3030–64. 10.1021/acsanm.1c03891 36568315 PMC9773423

[95] Marfavi Z Cai Y Lv Q Han Y Yang R Sun K The Synergy Between Antibiotics and the Nanoparticle-Based Photodynamic Effect. Nano Lett (2024). (in press). 10.1021/acs.nanolett.4c03668 39356053

[96] Qayyum S Oves M Khan AU . Obliteration of Bacterial Growth and Biofilm Through ROS Generation by Facilely Synthesized Green Silver Nanoparticles. PLoS One (2017) 12(8):e0181363. 10.1371/journal.pone.0181363 28771501 PMC5542591

[97] Jeyaraman M Jeyaraman N Nallakumarasamy A Iyengar KP Jain VK Potty AG Silver Nanoparticle Technology in Orthopaedic Infections. World J Orthop (2023) 14(9):662–8. 10.5312/wjo.v14.i9.662 37744720 PMC10514710

[98] Kalantari K Mostafavi E Afifi AM Izadiyan Z Jahangirian H Rafiee-Moghaddam R Wound Dressings Functionalized with Silver Nanoparticles: Promises and Pitfalls. Nanoscale (2020) 12(4):2268–91. 10.1039/c9nr08234d 31942896

[99] Yılmaz GE Göktürk I Ovezova M Yılmaz F Kılıç S Denizli A . Antimicrobial Nanomaterials: A Review. Hygiene (2023) 3:269–90. 10.3390/hygiene3030020

[100] Pino-Hurtado MS Fernández-Fernández R Torres C Robredo B . Searching for Antimicrobial-Producing Bacteria from Soils Through an Educational Project and Their Evaluation as Potential Biocontrol Agents. Antibiotics (Basel) (2023) 13(1):29. 10.3390/antibiotics13010029 38247588 PMC10812812

[101] Berdy B Spoering AL Ling LL Epstein SS . *In situ* Cultivation of Previously Uncultivable Microorganisms Using the Ichip. Nat Protoc (2017) 12(10):2232–42. 10.1038/nprot.2017.074 29532802

[102] Ling LL Schneider T Peoples AJ Spoering AL Engels I Conlon BP A New Antibiotic Kills Pathogens Without Detectable Resistance. Nature (2015) 517(7535):455–9. 10.1038/nature14098 25561178 PMC7414797

[103] Shukla R Lavore F Maity S Derks MGN Jones CR Vermeulen BJA Teixobactin Kills Bacteria by a Two-Pronged Attack on the Cell Envelope. Nature (2022) 608(7922):390–6. 10.1038/s41586-022-05019-y 35922513 PMC9365693

[104] Wright G Jangra M Travin D Aleksandrova E Kaur M Darwish L A Broad Spectrum Lasso Peptide Antibiotic Targeting the Bacterial Ribosome. Res Sq [Preprint]. (2024) Sep 16:rs.3.rs-5058118. 10.21203/rs.3.rs-5058118/v1 40140562 PMC12497486

[105] Jangra M Travin DY Aleksandrova EV Kaur M Darwish L Koteva K A Broad-Spectrum Lasso Peptide Antibiotic Targeting the Bacterial Ribosome. Nature (2025) 640(8060):1022–30. 10.1038/s41586-025-08723-7 40140562 PMC12497486

[106] Popoola PO Ijibadejo MS Adejumo DO Efeurhobo OD Ogunleye OO Olawumi LT Therapeutic Potentials of Phytochemicals in Combatting Antimicrobial Resistance (AMR). Asian J Res Biochem (2025) 15(1):48–62. 10.9734/ajrb/2025/v15i1350

[107] Anand U Jacobo-Herrera N Altemimi A Lakhssassi N . A Comprehensive Review on Medicinal Plants as Antimicrobial Therapeutics: Potential Avenues of Biocompatible Drug Discovery. Metabolites (2019) 9(11):258. 10.3390/metabo9110258 31683833 PMC6918160

[108] Millar BC Rao JR Moore JE . Fighting Antimicrobial Resistance (AMR): Chinese Herbal Medicine as a Source of Novel Antimicrobials - An Update. Lett Appl Microbiol (2021) 73(4):400–7. 10.1111/lam.13534 34219247

[109] Yang S Su P Li L Liu S Wang Y . Advances and Mechanisms of Traditional Chinese Medicine and Its Active Ingredients Against Antibiotic-Resistant *Escherichia coli* Infections. J Pharm Anal (2025) 15(2):101117. 10.1016/j.jpha.2024.101117 40026356 PMC11871446

[110] D'Almeida RE Molina RDI Viola CM Luciardi MC Nieto Peñalver C Bardón A Comparison of Seven Structurally Related Coumarins on the Inhibition of Quorum Sensing of *Pseudomonas aeruginosa* and *Chromobacterium violaceum* . Bioorg Chem (2017) 73:37–42. 10.1016/j.bioorg.2017.05.011 28599132

[111] Zuo GY Wang CJ Han J Li YQ Wang GC . Synergism of Coumarins from the Chinese Drug Zanthoxylum nitidum with Antibacterial Agents Against Methicillin-Resistant *Staphylococcus aureus* (MRSA). Phytomedicine (2016) 23(14):1814–20. 10.1016/j.phymed.2016.11.001 27912884

[112] Zainuddin ANZ Mustakim NN Rosemanzailani FA Fadilah NIM Maarof M Fauzi MB . A Comprehensive Review of Honey-Containing Hydrogel for Wound Healing Applications. Gels (2025) 11(3):194. 10.3390/gels11030194 40136899 PMC11942582

[113] Ogwu MC Izah SC . Honey as a Natural Antimicrobial. Antibiotics (Basel) (2025) 14(3):255. 10.3390/antibiotics14030255 40149066 PMC11939154

[114] Bava R Puteo C Lombardi R Garcea G Lupia C Spano A Antimicrobial Properties of Hive Products and Their Potential Applications in Human and Veterinary Medicine. Antibiotics (Basel) (2025) 14(2):172. 10.3390/antibiotics14020172 40001416 PMC11851452

[115] Ángyán VD Balázs VL Kocsis M Kocsis B Horváth G Farkas Á Synergistic Antibiofilm Effects of Chestnut and Linden Honey with Lavender Essential Oil Against Multidrug-Resistant Otitis Media Pathogens. Antibiotics (Basel) (2025) 14(2):146. 10.3390/antibiotics14020146 40001390 PMC11851655

[116] Girma A Seo W She RC . Antibacterial Activity of Varying UMF-Graded Manuka Honeys. PLoS One (2019) 14(10):e0224495. 10.1371/journal.pone.0224495 31652284 PMC6814216

[117] Abdellah F . Antimicrobial Properties of Natural Honey. In: Aziz MA , editor. Melittology - New Advances. London, United Kingdom: IntechOpen Limited (2023). 10.5772/intechopen.1003933

[118] Brudzynski K . Honey as an Ecological Reservoir of Antibacterial Compounds Produced by Antagonistic Microbial Interactions in Plant Nectars, Honey and Honey Bee. Antibiotics (Basel) (2021) 10(5):551. 10.3390/antibiotics10050551 34065141 PMC8151657

[119] Nolan VC Harrison J Cox JAG . Dissecting the Antimicrobial Composition of Honey. Antibiotics (Basel) (2019) 8(4):251. 10.3390/antibiotics8040251 31817375 PMC6963415

[120] Xiong ZR Sogin JH Worobo RW . Microbiome Analysis of Raw Honey Reveals Important Factors Influencing the Bacterial and Fungal Communities. Front Microbiol (2023) 13(13):1099522. 10.3389/fmicb.2022.1099522 36713191 PMC9877413

[121] Alluhaim W Alkhulaifi MM Alzahrani RR Alrfaei BM Yassin AEB Alghoribi MF Effectiveness of a Novel Liposomal Methylglyoxal-Tobramycin Formulation in Reducing Biofilm Formation and Bacterial Adhesion. Antibiotics (Basel) (2024) 14(1):3. 10.3390/antibiotics14010003 39858289 PMC11763214

[122] Mayekar VM Ali A Alim H Patel N . A Review: Antimicrobial Activity of the Medicinal Spice Plants to Cure Human Disease. Plant Sci Today (2021) 8(3):629–46. 10.14719/pst.2021.8.3.1152

[123] Moore RE Millar BC Panickar JR Moore JE . Interaction of South Asian Spices with Conventional Antibiotics: Implications for Antimicrobial Resistance for *Mycobacterium abscessus* and Cystic Fibrosis. Int J Mycobacteriol (2018) 7(3):257–60. 10.4103/ijmy.ijmy_72_18 30198506

[124] Vasconcelos NG Queiroz JHFS Silva KED Vasconcelos PCP Croda J Simionatto S . Synergistic Effects of Cinnamomum Cassia L. Essential Oil in Combination with Polymyxin B Against Carbapenemase-Producing *Klebsiella pneumoniae* and *Serratia marcescens* . PLoS One (2020) 15(7):e0236505. 10.1371/journal.pone.0236505 32701970 PMC7377461

[125] Seukep AJ Tamambang FM Matieta VY Mbuntcha HG Bomba FDT Kuete V Potential of Methanol Extracts of *Nephelium lappaceum* (*Sapindaceae*) and *Hyphaene thebaica* (*Arecaceae*) as Adjuvants to Enhance the Efficacy of Antibiotics Against Critical Class Priority Bacteria. PLoS One (2025) 20(2):e0314958. 10.1371/journal.pone.0314958 39937773 PMC11819497

[126] Ortega-Lozano AJ Hernández-Cruz EY Gómez-Sierra T Pedraza-Chaverri J . Antimicrobial Activity of Spices Popularly Used in Mexico Against Urinary Tract Infections. Antibiotics (Basel) (2023) 12(2):325. 10.3390/antibiotics12020325 36830236 PMC9952462

[127] Barbieri F Tabanelli G Braschi G Bassi D Morandi S Šimat V Mediterranean Plants and Spices as a Source of Bioactive Essential Oils for Food Applications: Chemical Characterisation and in Vitro Activity. Int J Mol Sci (2025) 26(8):3875. 10.3390/ijms26083875 40332532 PMC12027827

[128] El-Bilawy EH Mamdouh I Behiry S Teiba II . Evaluating the Antibacterial Efficacy of Bee Venom Against Multidrug-Resistant Pathogenic Bacteria: *Escherichia coli, Salmonella typhimurium*, and *Enterococcus faecalis* . World J Microbiol Biotechnol (2025) 41(2):40. 10.1007/s11274-024-04248-9 39821511 PMC11739217

[129] Teiba II Mazrou YSA Makhlouf AH Nehela Y Mohamed AE Abbas AM Antibacterial Potential of Honeybee Venom and *Monascus purpureus* Extracellular Metabolites Against Multidrug-Resistant Pathogenic Bacteria. Biology (Basel) (2024) 14(1):21. 10.3390/biology14010021 39857252 PMC11759185

[130] Wang K Mwangi J Cao K Wang Y Gao J Yang M Peptide Toxin Diversity and a Novel Antimicrobial Peptide from the Spider *Oxyopes forcipiformis* . Toxins (Basel) (2024) 16(11):466. 10.3390/toxins16110466 39591221 PMC11597926

[131] Edirisinghe EAHW Athukorala BN Perera M Abeywardana BASD Sigera PST Eranga P Jellyfish Venom Peptides Targeting Human Potassium Channels Identified Through Ligand Screening: Morphometric and Molecular Identification of the Species and Antibiotic Potential. Mar Drugs (2024) 22(8):333. 10.3390/md22080333 39195449 PMC11355547

[132] Nasr S Borges A Sahyoun C Nasr R Roufayel R Legros C Scorpion Venom as a Source of Antimicrobial Peptides: Overview of Biomolecule Separation, Analysis and Characterization Methods. Antibiotics (Basel) (2023) 12(9):1380. 10.3390/antibiotics12091380 37760677 PMC10525675

[133] Tawfik MM Bertelsen M Abdel-Rahman MA Strong PN Miller K . Scorpion Venom Antimicrobial Peptides Induce Siderophore Biosynthesis and Oxidative Stress Responses in *Escherichia coli* . mSphere (2021) 6(3):e00267–21. 10.1128/mSphere.00267-21 33980680 PMC8125054

[134] Zhu C Zhao Y Zhao X Liu S Xia X Zhang S The Antimicrobial Peptide MPX Can Kill *Staphylococcus aureus*, Reduce Biofilm Formation, and Effectively Treat Bacterial Skin Infections in Mice. Front Vet Sci (2022) 9:819921. 10.3389/fvets.2022.819921 35425831 PMC9002018

[135] Fratini F Cilia G Turchi B Felicioli A . Insects, Arachnids and Centipedes Venom: A Powerful Weapon Against Bacteria. A Literature Review. Toxicon (2017) 130:91–103. 10.1016/j.toxicon.2017.02.020 28242227

[136] Nunes E Frihling B Barros E de Oliveira C Verbisck N Flores T Antibiofilm Activity of Acidic Phospholipase Isoform Isolated from *Bothrops erythromelas* Snake Venom. Toxins (Basel) (2020) 12(9):606. 10.3390/toxins12090606 32962193 PMC7551604

[137] Khan NA Amorim FG Dunbar JP Leonard D Redureau D Quinton L Inhibition of Bacterial Biofilms by the Snake Venom Proteome. Biotechnol Rep (Amst) (2023) 39:e00810. 10.1016/j.btre.2023.e00810 37559690 PMC10407894

[138] Figueroa-Montiel A Bernáldez J Jiménez S Ueberhide B González LJ Licea-Navarro A . Antimycobacterial Activity: A New Pharmacological Target for Conotoxins Found in the First Reported Conotoxin from *Conasprella ximenes* . Toxins (Basel) (2018) 10(2):51. 10.3390/toxins10020051 29360782 PMC5848152

[139] Quinn GA Dyson PJ . Going to Extremes: Progress in Exploring New Environments for Novel Antibiotics. NPJ Antimicrob Resist (2024) 2(1):8. 10.1038/s44259-024-00025-8 39843508 PMC11721673

[140] Scambos TA Campbell GG Pope A Haran T Muto A Lazzara M Ultralow Surface Temperatures in East Antarctica from Satellite Thermal Infrared Mapping: The Coldest Places on Earth. Geophys Res Lett (2018) 45(12):6124–33. 10.1029/2018GL078133

[141] Núñez-Montero K Barrientos L . Advances in Antarctic Research for Antimicrobial Discovery: A Comprehensive Narrative Review of Bacteria from Antarctic Environments as Potential Sources of Novel Antibiotic Compounds Against Human Pathogens and Microorganisms of Industrial Importance. Antibiotics (Basel) (2018) 7(4):90. 10.3390/antibiotics7040090 30347637 PMC6316688

[142] de França P Costa JH Fill TP Lancellotti M Ruiz ALTG Fantinatti-Garboggini F . Genome Mining Reveals Secondary Metabolites of Antarctic Bacterium *Streptomyces albidoflavus* Related to Antimicrobial and Antiproliferative Activities. Arch Microbiol (2023) 205(11):354. 10.1007/s00203-023-03691-w 37828121

[143] Yang F Sang M Lu JR Zhao HM Zou Y Wu W Somalactams A-D: Anti-Inflammatory Macrolide Lactams with Unique Ring Systems from an Arctic *Actinomycete* Strain. Angew Chem Int Ed Engl (2023) 62(18):e202218085. 10.1002/anie.202218085 36680430

[144] Silva MB Feitosa AO Lima IGO Bispo JRS Santos ACM Moreira MSA Antarctic Organisms as a Source of Antimicrobial Compounds: A Patent Review. An Acad Bras Cienc (2022) 94(Suppl. 1):e20210840. 10.1590/0001-3765202220210840 35384978

[145] Imperlini E Massaro F Grifoni A Maiurano F Taddei AR Borocci S Membrane Alteration, Anti-Virulence Properties and Metabolomic Perturbation of a Chionodracine-Derived Antimicrobial Peptide, KHS-Cnd, on Two Bacteria Models. Peptides (2024) 82:171311. 10.1016/j.peptides.2024.171311 39426570

[146] Della Pelle G Perà G Belardinelli MC Gerdol M Felli M Crognale S Trematocine, a Novel Antimicrobial Peptide from the Antarctic Fish *Trematomus Bernacchii*: Identification and Biological Activity. Antibiotics (Basel) (2020) 9(2):66. 10.3390/antibiotics9020066 32041161 PMC7168153

[147] Squitieri D Massaro F Graziano MM Borocci S Cacaci M Di Vito M Trematocine-Derived Antimicrobial Peptides from the Antarctic Fish *Trematomus bernacchaii*: Potent Antibacterial Agents Against ESKAPE Pathogens. Front Microbiol (2024) 15:1447301. 10.3389/fmicb.2024.1447301 39171261 PMC11335685

[148] Fei Y Wang Q Lu J Ouyang L Li W Hu R Identification of Antibacterial Activity of LEAP2 from Antarctic Icefish *Chionodraco Hamatus* . J Fish Dis (2023) 46(9):905–16. 10.1111/jfd.13797 37245215

[149] Shin SC Ahn IH Ahn DH Lee YM Lee J Lee JH Characterization of Two Antimicrobial Peptides from Antarctic Fishes (*Notothenia coriiceps* and *Parachaenichthys charcoti*). PLoS One (2017) 12(1):e0170821. 10.1371/journal.pone.0170821 28122029 PMC5266299

[150] Xiao Y Yan F Cui Y Du J Hu G Zhai W A Symbiotic Bacterium of Antarctic Fish Reveals Environmental Adaptability Mechanisms and Biosynthetic Potential Towards Antibacterial and Cytotoxic Activities. Front Microbiol (2023) 13:1085063. 10.3389/fmicb.2022.1085063 36713225 PMC9882997

[151] Du Y Han W Hao P Hu Y Hu T Zeng Y . A Genomics-Based Discovery of Secondary Metabolite Biosynthetic Gene Clusters in the Potential Novel Strain *Streptomyces* sp. 21So2-11 Isolated from Antarctic Soil. Microorganisms (2024) 12(6):1228. 10.3390/microorganisms12061228 38930610 PMC11205464

[152] D'Angelo C Trecca M Carpentieri A Artini M Selan L Tutino ML Cold-Azurin, a New Antibiofilm Protein Produced by the Antarctic Marine Bacterium *Pseudomonas* sp. TAE6080. Mar Drugs (2024) 22(2):61. 10.3390/md22020061 38393032 PMC10890351

[153] Camacho KF de Melo Carlos L Bernal SPF de Oliveira VM Ruiz JLM Ottoni JR Antarctic Marine Sediment as a Source of Filamentous Fungi-Derived Antimicrobial and Antitumor Compounds of Pharmaceutical Interest. Extremophiles (2024) 28(2):21. 10.1007/s00792-024-01339-1 38532228

[154] Artini M Papa R Vrenna G Trecca M Paris I D'Angelo C Antarctic Marine Bacteria as a Source of Anti-Biofilm Molecules to Combat ESKAPE Pathogens. Antibiotics (Basel) (2023) 12(10):1556. 10.3390/antibiotics12101556 37887257 PMC10604463

[155] D'Angelo C Casillo A Melchiorre C Lauro C Corsaro MM Carpentieri A CATASAN Is a New Anti-Biofilm Agent Produced by the Marine Antarctic Bacterium *Psychrobacter* sp. TAE2020. Mar Drugs (2022) 20(12):747. 10.3390/md20120747 36547894 PMC9785100

[156] Nikitin DA Sadykova VS Kuvarina AE Dakh AG Biryukov MV . Enzymatic and Antimicrobial Activities in Polar Strains of Microscopic Soil Fungi. Dokl Biol Sci (2022) 507(1):380–93. 10.1134/S0012496622060151 36781534

[157] Saleem M Hassan A Li F Lu Q Ponomareva LV Parkin S Bioprospecting of Desert Actinobacteria with Special Emphases on Griseoviridin, Mitomycin C and a New Bacterial Metabolite Producing *Streptomyces* sp. PU-KB10-4. BMC Microbiol (2023) 23(1):69. 10.1186/s12866-023-02770-8 36922786 PMC10015687

[158] Yekkour A Meklat A Bijani C Toumatia O Errakhi R Lebrihi A A Novel Hydroxamic Acid-Containing Antibiotic Produced by a Saharan Soil-Living S*treptomyces* Strain. Lett Appl Microbiol (2015) 60(6):589–96. 10.1111/lam.12412 25754683

[159] Jiang ZK Guo L Chen C Liu SW Zhang L Dai SJ Xiakemycin A, a Novel Pyranonaphthoquinone Antibiotic, Produced by the *Streptomyces* sp. CC8-201 from the Soil of a Karst Cave. J Antibiot (Tokyo) (2015) 68(12):771–4. 10.1038/ja.2015.70 26104142

[160] Saeed A Modafer Y Ageeli AA Madkhli AY Pashameah RA Al-Marhaby FA Harnessing Volcanic Silica Nanoparticles for Antibacterial Applications. Environ Technology and Innovation (2023) 30:103111. 10.1016/j.eti.2023.103111

[161] Gutiérrez-Almada K González-Acosta B Borges-Souza JM Aguila-Ramírez RN . Marine Bacteria Associated with Shallow Hydrothermal Systems in the Gulf of California with the Capacity to Produce Biofilm Inhibiting Compounds. Arch Microbiol (2020) 202(6):1477–88. 10.1007/s00203-020-01851-w 32193579

[162] Keeler E Burgaud G Teske A Beaudoin D Mehiri M Dayras M Deep-Sea Hydrothermal Vent Sediments Reveal Diverse Fungi with Antibacterial Activities. FEMS Microbiol Ecol (2021) 97(8):fiab103. 10.1093/femsec/fiab103 34245561

[163] Wang Y Zhang J Sun Y Sun L . A Crustin from Hydrothermal Vent Shrimp: Antimicrobial Activity and Mechanism. Mar Drugs (2021) 19(3):176. 10.3390/md19030176 33807037 PMC8005205

[164] Pardo-Esté C Cortés J Castro-Severyn J Pérez V Henriquez-Aedo K Cuadros F Secondary Metabolites with Antimicrobial Activity Produced by Thermophilic Bacteria from a High-Altitude Hydrothermal System. Front Microbiol (2024) 15:1477458. 10.3389/fmicb.2024.1477458 39411441 PMC11474921

[165] Thompson TP Gilmore BF . Exploring Halophilic Environments as a Source of New Antibiotics. Crit Rev Microbiol (2024) 50(3):341–70. 10.1080/1040841X.2023.2197491 37079280

[166] Zubair M Fatima F Rahman S Alrasheed T Alatawy R Mesaik MA . Disruption of Biofilm Formation by Dead Sea Soil Extracts: A Novel Approach Against Diabetic Foot Wound Isolates. Microbiol Res (2024) 15:2535–54. 10.3390/microbiolres15040169

[167] Hitchcock NM Devequi Gomes Nunes D Shiach J Valeria Saraiva Hodel K Dantas Viana Barbosa J Alencar PRL Current Clinical Landscape and Global Potential of Bacteriophage Therapy. Viruses (2023) 15(4):1020. 10.3390/v15041020 37113000 PMC10146840

[168] Nabi-Afjadi M Teymouri S Monfared FN Varnosfaderani SMN Halimi H . The Human Gut Phageome: Identification and Roles in the Diseases. J Cell Signal (2023) 4(3):128–41. 10.33696/Signaling.4.100

[169] Schooley RT Biswas B Gill JJ Hernandez-Morales A Lancaster J Lessor L Development and Use of Personalized Bacteriophage-Based Therapeutic Cocktails to Treat a Patient with a Disseminated Resistant *Acinetobacter baumannii* Infection. Antimicrob Agents Chemother (2017) 61(10):e00954–17. 10.1128/AAC.00954-17 28807909 PMC5610518

[170] Aslam S Lampley E Wooten D Karris M Benson C Strathdee S Lessons Learned from the First 10 Consecutive Cases of Intravenous Bacteriophage Therapy to Treat Multidrug-Resistant Bacterial Infections at a Single Center in the United States. Open Forum Infect Dis (2020) 7(9):ofaa389. 10.1093/ofid/ofaa389 33005701 PMC7519779

[171] Khosravi A Chen Q Echterhof A Koff JL Bollyky PL . Phage Therapy for Respiratory Infections: Opportunities and Challenges. Lung (2024) 202(3):223–32. 10.1007/s00408-024-00700-7 38772946 PMC11570333

[172] Health Improvement Scotland. Bacteriophage Therapy for Patients with Difficult to Treat Bacterial Infections (2023). Available online at: https://shtg.scot/our-advice/bacteriophage-therapy-for-patients-with-difficult-to-treat-bacterial-infections/ (Accessed July 21, 2025).

[173] The Science, Innovation and Technology Select Committee. House of Commons, UK Parliament. In: The Antimicrobial Potential of Bacteriophages (2024). Available online at: https://publications.parliament.uk/pa/cm5804/cmselect/cmsctech/328/report.html (Accessed July 21, 2025).

[174] Department of Health and Social Care. Policy Paper: Government's Response to the Science, Innovation and Technology Committee's Report 'The Antimicrobial Potential of Bacteriophages'. London, United Kingdom: Department of Health & Social Care (2024). Available online at: https://www.gov.uk/government/publications/the-antimicrobial-potential-of-bacteriophages-report-government-response/governments-response-to-the-science-innovation-and-technology-committees-report-the-antimicrobial-potential-of-bacteriophages (Accessed July 21, 2025).

[175] Jones JD Trippett C Suleman M Clokie MRJ Clark JR . The Future of Clinical Phage Therapy in the United Kingdom. Viruses (2023) 15(3):721. 10.3390/v15030721 36992430 PMC10053292

[176] Polat E Kang K . Natural Photosensitizers in Antimicrobial Photodynamic Therapy. Biomedicines (2021) 9(6):584. 10.3390/biomedicines9060584 34063973 PMC8224061

[177] Songca SP Adjei Y . Applications of Antimicrobial Photodynamic Therapy Against Bacterial Biofilms. Int J Mol Sci (2022) 23(6):3209. 10.3390/ijms23063209 35328629 PMC8953781

[178] Mathur A Parihar AS Modi S Kalra A . Photodynamic Therapy for ESKAPE Pathogens: An Emerging Approach to Combat Antimicrobial Resistance (AMR). Microb Pathog (2023) 183:106307. 10.1016/j.micpath.2023.106307 37604213

[179] Piksa M Lian C Samuel IC Pawlik KJ Samuel IDW Matczyszyn K . The Role of the Light Source in Antimicrobial Photodynamic Therapy. Chem Soc Rev (2023) 52(5):1697–722. 10.1039/d0cs01051k 36779328

[180] Sellera FP Sabino CP Núñez SC Ribeiro MS . Clinical Acceptance of Antimicrobial Photodynamic Therapy in the Age of WHO Global Priority Pathogens: So what We Need to Move Forward? Photodiagnosis Photodyn Ther (2022) 40:103158. 10.1016/j.pdpdt.2022.103158 36244682 PMC9558772

[181] Alves F Stringasci MD Requena MB Blanco KC Dias LD Corrêa TQ Randomized and Controlled Clinical Studies on Antibacterial Photodynamic Therapy: An Overview. Photonics (2022) 9(5):340. 10.3390/photonics9050340

[182] Lin J Cao M Wang S Wu X Pan Y Dai Z Deep Red-Light-Mediated Nitric Oxide and Photodynamic Synergistic Antibacterial Therapy for the Treatment of Drug-Resistant Bacterial Infections. Small (2025) 21(6):e2408759. 10.1002/smll.202408759 39780624

[183] de Freitas AB Rezende HHA de Souza GRL Gonçalves PJ . Photodynamic Inactivation of KPC-Producing *Klebsiella pneumoniae* Difficult-to-Treat Resistance (DTR) by a Cationic Porphyrin. J Photochem Photobiol B (2025) 265:113133. 10.1016/j.jphotobiol.2025.113133 39987860

[184] Woźniak A Grinholc M . Combined Antimicrobial Blue Light and Antibiotics as a Tool for Eradication of Multidrug-Resistant Isolates of *Pseudomonas aeruginosa* and *Staphylococcus aureus*: In Vitro and In Vivo Studies. Antioxidants (Basel) (2022) 11(9):1660. 10.3390/antiox11091660 36139734 PMC9495928

[185] Shiralizadeh S Farmani A Shokoohizadeh L Pourhajibagher M Alikhani MY Bahador A . The Effect of Photodynamic Therapy Using Gentamicin and Imipenem-Derived Carbon Dots on *Pseudomonas aeruginosa Isolates* . Lasers Med Sci (2025) 40(1):150. 10.1007/s10103-025-04402-1 40105985

[186] Boluki E Kazemian H Peeridogaheh H Alikhani MY Shahabi S Beytollahi L Antimicrobial Activity of Photodynamic Therapy in Combination with Colistin Against a Pan-Drug Resistant *Acinetobacter baumannii* Isolated from Burn Patient. Photodiagnosis Photodyn Ther (2017) 18:1–5. 10.1016/j.pdpdt.2017.01.003 28088439

[187] Chibebe Junior J Fuchs BB Sabino CP Junqueira JC Jorge AO Ribeiro MS Photodynamic and Antibiotic Therapy Impair the Pathogenesis *of Enterococcus faecium* in a Whole Animal Insect Model. PLoS One (2013) 8(2):e55926. 10.1371/journal.pone.0055926 23457486 PMC3573038

[188] da Cunha IV da Silva Oliveira DD Calefi GG Silva NBS Martins CHG Rezende Júnior CO Photosensitizer Associated with Efflux Pump Inhibitors as a Strategy for Photodynamic Therapy Against Bacterial Resistance. Eur J Med Chem (2025) 284:117197. 10.1016/j.ejmech.2024.117197 39731789

[189] Allamyradov Y Yosef JB Kylychbekov S Majidov I Khuzhakulov Z Er AY The Role of Efflux Pump Inhibitor in Enhancing Antimicrobial Efficiency of Ag NPs and MB as an Effective Photodynamic Therapy Agent. Photodiagnosis Photodyn Ther (2024) 47:104212. 10.1016/j.pdpdt.2024.104212 38740317

[190] Shirdel Z Fekrirad Z . Efflux Pump Inhibitor Potentiates the Antimicrobial Photodynamic Inactivation of Multidrug-Resistant *Acinetobacter baumannii* . Photobiomodul Photomed Laser Surg (2024) 42(4):314–20. 10.1089/photob.2023.0194 38536111

[191] Surur AK de Santana RL Palhares AL de Oliveira AB De Annunzio SR de Souza de Santana WMO Exploring Catalase Inhibition as an Adjuvant to Antimicrobial Photodynamic Therapy Against *Staphylococcus aureus* . Photochem Photobiol Sci (2025) 24(6):1053–68. 10.1007/s43630-025-00735-6 40410636

[192] Pourhajibagher M Bahrami R Bahador A . Application of Antimicrobial Sonodynamic Therapy as a Potential Treatment Modality in Dentistry: A Literature Review. J Dent Sci (2024) 19(2):787–94. 10.1016/j.jds.2023.11.006 38618114 PMC11010677

[193] Fan L Idris Muhammad A Bilyaminu Ismail B Liu D . Sonodynamic Antimicrobial Chemotherapy: An Emerging Alternative Strategy for Microbial Inactivation. Ultrason Sonochem (2021) 75:105591. 10.1016/j.ultsonch.2021.105591 34082219 PMC8182071

[194] Mu WB Yao LQ Guo ZY Ma YC Wang F Yang JH . Enhancing Biofilm Disruption and Bactericidal Efficiency Using Vancomycin-Loaded Microbubbles in Sonodynamic Therapy. JAC Antimicrob Resist (2025) 7(2):dlaf045. 10.1093/jacamr/dlaf045 40110553 PMC11920867

[195] Song X Li J Huang S Zhang Y Hong S Zhang X . A Facile Coordination Polymer Nanoparticle for Sonodynamic Therapy Combating Drug-Resistant Bacteria. Small (2025) 30:e2501131. 10.1002/smll.202501131 40445185

[196] Okda M Spina S Safaee Fakhr B Carroll RW . The Antimicrobial Effects of Nitric Oxide: A Narrative Review. Nitric Oxide (2025) 155:20–40. 10.1016/j.niox.2025.01.001 39793728

[197] Webster CM Shepherd M . The Nitric Oxide Paradox: Antimicrobial and Inhibitor of Antibiotic Efficacy. *Emerg Top Life Sc*i (2024) 8(1):37–43. 10.1042/ETLS20230114 37975610 PMC10903473

[198] Deppisch C Herrmann G Graepler-Mainka U Wirtz H Heyder S Engel C Gaseous Nitric Oxide to Treat Antibiotic Resistant Bacterial and Fungal Lung Infections in Patients with Cystic Fibrosis: A Phase I Clinical Study. Infection (2016) 44(4):513–20. 10.1007/s15010-016-0879-x 26861246

[199] Howlin RP Cathie K Hall-Stoodley L Cornelius V Duignan C Allan RN Low-Dose Nitric Oxide as Targeted Anti-Biofilm Adjunctive Therapy to Treat Chronic *Pseudomonas aeruginosa* Infection in Cystic Fibrosis. Mol Ther (2017) 25(9):2104–16. 10.1016/j.ymthe.2017.06.021 28750737 PMC5589160

[200] Bentur L Gur M Ashkenazi M Livnat-Levanon G Mizrahi M Tal A Pilot Study to Test Inhaled Nitric Oxide in Cystic Fibrosis Patients with Refractory *Mycobacterium abscessus* Lung Infection. J Cyst Fibros (2020) 19(2):225–31. 10.1016/j.jcf.2019.05.002 31129068

[201] Bartley BL Gardner KJ Spina S Hurley BP Campeau D Berra L High-Dose Inhaled Nitric Oxide as Adjunct Therapy in Cystic Fibrosis Targeting *Burkholderia multivorans* . Case Rep Pediatr (2020) 2020:1536714. 10.1155/2020/1536714 32685229 PMC7334765

[202] Flume PA Garcia BA Wilson D Steed L Dorman SE Winthrop K . Inhaled Nitric Oxide for Adults with Pulmonary NonTuberculous Mycobacterial Infection. Respir Med (2023) 206:107069. 10.1016/j.rmed.2022.107069 36493605

[203] Millar BC Moore JE . Antimycobacterial Strategies to Evade Antimicrobial Resistance in the Nontuberculous Mycobacteria. Int J Mycobacteriol (2019) 8(1):7–21. 10.4103/ijmy.ijmy_153_18 30860173

[204] Tortella FG Fincheira P Rubilar O Leiva S Fernandez I Schoebitz M Nanoparticle-Based Nitric Oxide Donors: Exploring Their Antimicrobial and Anti-Biofilm Capabilities. Antibiotics (Basel) (2024) 13(11):1047. 10.3390/antibiotics13111047 39596741 PMC11591520

[205] Food and Drug Administration. Rebyota (2022). Available online at: https://www.fda.gov/vaccines-blood-biologics/vaccines/rebyota (Accessed July 01, 2025).

[206] Khanna S Assi M Lee C Yoho D Louie T Knapple W Efficacy and Safety of RBX2660 in PUNCH CD3, a Phase III, Randomized, Double-Blind, Placebo-Controlled Trial with a Bayesian Primary Analysis for the Prevention of Recurrent *Clostridioides difficile* Infection. Drugs (2022) 82(15):1527–38. 10.1007/s40265-022-01797-x 36287379 PMC9607700

[207] Food and Drug Administration. FDA Approves First Orally Administered Fecal Microbiota Product for the Prevention of Recurrence of *Clostridioides difficile* Infection (2023). Available online at: https://www.fda.gov/news-events/press-announcements/fda-approves-first-orally-administered-fecal-microbiota-product-prevention-recurrence-clostridioides (Accessed July 01, 2025).

[208] Tixier EN Verheyen E Ungaro RC Grinspan AM . Faecal Microbiota Transplant Decreases Mortality in Severe and Fulminant Clostridioides difficile Infection in Critically Ill Patients. Aliment Pharmacol Ther (2019) 50(10):1094–9. 10.1111/apt.15526 31612528 PMC6817391

[209] Dongre DS Saha UB Saroj SD . Exploring the Role of Gut Microbiota in Antibiotic Resistance and Prevention. Ann Med (2025) 57(1):2478317. 10.1080/07853890.2025.2478317 40096354 PMC11915737

[210] Pérez-Nadales E Cano Á Recio M Artacho MJ Guzmán-Puche J Doblas A Randomised, Double-Blind, Placebo-Controlled, Phase 2, Superiority Trial to Demonstrate the Effectiveness of Faecal Microbiota Transplantation for Selective Intestinal Decolonisation of Patients Colonised by Carbapenemase-Producing *Klebsiella pneumoniae* (KAPEDIS). BMJ Open (2022) 12(4):e058124. 10.1136/bmjopen-2021-058124 35387830 PMC8987760

[211] Saha S Tariq R Tosh PK Pardi DS Khanna S . Faecal Microbiota Transplantation for Eradicating Carriage of Multidrug-Resistant Organisms: A Systematic Review. Clin Microbiol Infect (2019) 25(8):958–63. 10.1016/j.cmi.2019.04.006 30986562

[212] Bajaj JS Shamsaddini A Fagan A Sterling RK Gavis E Khoruts A Fecal Microbiota Transplant in Cirrhosis Reduces Gut Microbial Antibiotic Resistance Genes: Analysis of Two Trials. Hepatol Commun (2020) 5(2):258–71. 10.1002/hep4.1639 33553973 PMC7850310

[213] Ghani R Mullish BH McDonald JAK Ghazy A Williams HRT Brannigan ET Disease Prevention Not Decolonization: A Model for Fecal Microbiota Transplantation in Patients Colonized with Multidrug-Resistant Organisms. Clin Infect Dis (2021) 72(8):1444–7. 10.1093/cid/ciaa948 32681643 PMC8075030

[214] Woodworth MH Conrad RE Haldopoulos M Pouch SM Babiker A Mehta AK Fecal Microbiota Transplantation Promotes Reduction of Antimicrobial Resistance by Strain Replacement. Sci Transl Med (2023) 15(720):eabo2750. 10.1126/scitranslmed.abo2750 37910603 PMC10821315

[215] Biliński J Grzesiowski P Muszyński J Wróblewska M Mądry K Robak K Fecal Microbiota Transplantation Inhibits Multidrug-Resistant Gut Pathogens: Preliminary Report Performed in an Immunocompromised Host. Arch Immunol Ther Exp (Warsz) (2016) 64(3):255–8. 10.1007/s00005-016-0387-9 26960790 PMC4863031

[216] Su F Luo Y Yu J Shi J Zhao Y Yan M Tandem Fecal Microbiota Transplantation Cycles in an Allogeneic Hematopoietic Stem Cell Transplant Recipient Targeting Carbapenem-Resistant *Enterobacteriaceae* Colonization: A Case Report and Literature Review. Eur J Med Res (2021) 26(1):37. 10.1186/s40001-021-00508-8 33910622 PMC8080403

[217] Innes AJ Mullish BH Ghani R Szydlo RM Apperley JF Olavarria E Fecal Microbiota Transplant Mitigates Adverse Outcomes Seen in Patients Colonized with Multidrug-Resistant Organisms Undergoing Allogeneic Hematopoietic Cell Transplantation. *Front Cell Infect Microbio*l (2021) 11:684659. 10.3389/fcimb.2021.684659 34513724 PMC8430254

[218] Nooij S Vendrik KEW Zwittink RD Ducarmon QR Keller JJ Kuijper EJ Long-Term Beneficial Effect of Faecal Microbiota Transplantation on Colonisation of Multidrug-Resistant Bacteria and Resistome Abundance in Patients with Recurrent *Clostridioides difficile Infection* . Genome Med (2024) 16(1):37. 10.1186/s13073-024-01306-7 38419010 PMC10902993

[219] Grosen AK Povlsen JV Lemming LE Jørgensen SMD Dahlerup JF Hvas CL . Faecal Microbiota Transplantation Eradicated Extended-Spectrum Beta-Lactamase-Producing *Klebsiella pneumoniae* from a Renal Transplant Recipient with Recurrent Urinary Tract Infections. Case Rep Nephrol Dial (2019) 9(2):102–7. 10.1159/000502336 31559265 PMC6751418

[220] Dharmaratne P Rahman N Leung A Ip M . Is There a Role of Faecal Microbiota Transplantation in Reducing Antibiotic Resistance Burden in Gut? A Systematic Review and Meta-Analysis. Ann Med (2021) 53(1):662–81. 10.1080/07853890.2021 34170204 PMC8238059

[221] Food and Drug Administration. Safety Alert: Fecal Microbiota for Transplantation and Risk of Transmission of Multi-Drug Resistant Organisms (2019). Available online at: https://www.cancerhealth.com/blog/safety-alert-fecal-microbiota-transplantation-risk-transmission-multidrug-resistant-organisms (Accessed July 01, 2025).

[222] Kelly CR Laine LA Wu GD . Monitoring Fecal Microbiota Transplantation Practice in a Rapidly Evolving Health and Regulatory Environment. Gastroenterology (2020) 159(6):2004–6. 10.1053/j.gastro.2020.08.039 32841646 PMC7443160

[223] Yadegar A Bar-Yoseph H Monaghan TM Pakpour S Severino A Kuijper EJ Fecal Microbiota Transplantation: Current Challenges and Future Landscapes. Clin Microbiol Rev (2024) 37(2):e0006022. 10.1128/cmr.00060-22 38717124 PMC11325845

[224] Grigoryan Z Shen MJ Twardus SW Beuttler MM Chen LA Bateman-House A . Fecal Microbiota Transplantation: Uses, Questions, and Ethics. Med Microecol (2020) 6:100027. 10.1016/j.medmic.2020.100027 33834162 PMC8026161

[225] Murray TS Herbst J . The Ethics of Fecal Microbiota Transplant as a Tool for Antimicrobial Stewardship Programs. J L Med Ethics (2019) 47(4):541–54. 10.1177/1073110519897730 31957576

[226] Bilsen MP Lambregts MMC van Prehn J Kuijper EJ . Faecal Microbiota Replacement to Eradicate Antimicrobial Resistant Bacteria in the Intestinal Tract - a Systematic Review. Curr Opin Gastroenterol (2022) 38(1):15–25. 10.1097/MOG.0000000000000792 34636363 PMC8654246

[227] Macareño-Castro J Solano-Salazar A Dong LT Mohiuddin M Espinoza JL . Fecal Microbiota Transplantation for Carbapenem-Resistant *Enterobacteriaceae*: A Systematic Review. J Infect (2022) 84(6):749–59. 10.1016/j.jinf.2022.04.028 35461908

[228] Atterbury RJ Tyson J . Predatory Bacteria as Living Antibiotics - where Are We Now? Microbiology (Reading) (2021) 167(1):167. 10.1099/mic.0.001025 33465024

[229] Cavallo FM Jordana L Friedrich AW Glasner C van Dijl JM . *Bdellovibrio Bacteriovorus*: A Potential 'Living Antibiotic' to Control Bacterial Pathogens. Crit Rev Microbiol (2021) 47(5):630–46. 10.1080/1040841X.2021.1908956 33934682

[230] Mahboub H Almanaa TN Al AA Rana MF Elabd H . Bacteriocin and Predatory Bacteria. In: Elumalai P Lakshmi S , editors. Antimicrobial Resistance in Aquaculture and Aquatic Environments. Singapore: Springer (2025). 10.1007/978-981-97-7320-6_11

[231] George S Sharma RK Hazarika R Hazarika S Ahmed R Phangchopi D . Predatory Bacteria: A Novel Approach to Antibiotic Substitution. Int J Bio-Resource Stress Management (2023) 14(1):045–53. 10.23910/1.2023.3168

[232] Mun W Upatissa S Lim S Dwidar M Mitchell RJ . Outer Membrane Porin F in *E. coli* Is Critical for Effective Predation by *Bdellovibrio* . Microbiol Spectr (2022) 10(6):e0309422. 10.1128/spectrum.03094-22 36445149 PMC9769668

[233] Romanowski EG Brothers KM Calvario RC Stella NA Kim T Elsayed M Predatory Bacteria Prevent the Proliferation of Intraocular *Serratia marcescens* and Fluoroquinolone-Resistant *Pseudomonas aeruginosa* . Microbiology (Reading) (2024) 170(2):001433. 10.1099/mic.0.001433 38358321 PMC10924457

[234] Saralegui C Herencias C Halperin AV de Dios-Caballero J Pérez-Viso B Salgado S Strain-Specific Predation of *Bdellovibrio bacteriovorus* on *Pseudomonas aeruginosa* with a Higher Range for Cystic Fibrosis than for Bacteremia Isolates. Sci Rep (2022) 12(1):10523. 10.1038/s41598-022-14378-5 35732651 PMC9217795

[235] McCulloch TR Wells TJ Souza-Fonseca-Guimaraes F . Towards Efficient Immunotherapy for Bacterial Infection. Trends Microbiol (2022) 30(2):158–69. 10.1016/j.tim.2021.05.005 34253452

[236] Odoom A Osman AH Dzuvor CKO . Recent Advances in Immunotherapeutic and Vaccine-Based Approaches for the Treatment of Drug-Resistant Bacterial Infections. ACS Infect Dis (2025) 11(6):1366–402. 10.1021/acsinfecdis.5c00001 40315159

[237] Frost I Sati H Garcia-Vello P Hasso-Agopsowicz M Lienhardt C Gigante V The Role of Bacterial Vaccines in the Fight Against Antimicrobial Resistance: An Analysis of the Preclinical and Clinical Development Pipeline. Lancet Microbe (2023) 4(2):e113–e125. 10.1016/S2666-5247(22)00303-2 36528040 PMC9892012

[238] World Health Organization. Estimating the Impact of Vaccines in Reducing Antimicrobial Resistance and Antibiotic Use: Technical Report (2024). Available online at: https://www.who.int/publications/i/item/9789240098787 (Accessed July 01, 2025).

[239] Cesaro A Hoffman SC Das P de la Fuente-Nunez C . Challenges and Applications of Artificial Intelligence in Infectious Diseases and Antimicrobial Resistance. NPJ Antimicrob Resist (2025) 3(1):2. 10.1038/s44259-024-00068-x 39843587 PMC11721440

[240] Stokes JM Yang K Swanson K Jin W Cubillos-Ruiz A Donghia NM A Deep Learning Approach to Antibiotic Discovery. Cell (2020) 180(4):688–702.e13. 10.1016/j.cell.2020.01.021 32084340 PMC8349178

[241] Rahman ASMZ Liu C Sturm H Hogan AM Davis R Hu P A Machine Learning Model Trained on a High-Throughput Antibacterial Screen Increases the Hit Rate of Drug Discovery. Plos Comput Biol (2022) 18(10):e1010613. 10.1371/journal.pcbi.1010613 36228001 PMC9624395

[242] Nasution AK Alqaaf M Islam RM Wijaya SH Ono N Kanaya S Identifying Potential Natural Antibiotics from Unani Formulas Through Machine Learning Approaches. Antibiotics (Basel) (2024) 13(10):971. 10.3390/antibiotics13100971 39452237 PMC11504695

[243] Lane T Russo DP Zorn KM Clark AM Korotcov A Tkachenko V Comparing and Validating Machine Learning Models for *Mycobacterium tuberculosis* Drug Discovery. Mol Pharm (2018) 15(10):4346–60. 10.1021/acs.molpharmaceut.8b00083 29672063 PMC6167198

[244] Schmalstig AA Zorn KM Murcia S Robinson A Savina S Komarova E *Mycobacterium abscessus* Drug Discovery Using Machine Learning. Tuberculosis (Edinb) (2022) 132:102168. 10.1016/j.tube.2022.102168 35077930 PMC8855326

[245] Li Y Cui X Yang X Liu G Zhang J . Artificial Intelligence in Predicting Pathogenic Microorganisms' Antimicrobial Resistance: Challenges, Progress, and Prospects. Front Cell Infect Microbiol (2024) 14:1482186. 10.3389/fcimb.2024.1482186 39554812 PMC11564165

[246] Arnold A McLellan S Stokes JM . How AI Can Help Us Beat AMR. NPJ Antimicrob Resist (2025) 3(1):18. 10.1038/s44259-025-00085-4 40082590 PMC11906734

[247] Liu GY Yu D Fan MM Zhang X Jin ZY Tang C Antimicrobial Resistance Crisis: Could Artificial Intelligence Be the Solution? Mil Med Res (2024) 11(1):7. 10.1186/s40779-024-00510-1 38254241 PMC10804841

[248] Bilal H Khan MN Khan S Shafiq M Fang W Khan RU The Role of Artificial Intelligence and Machine Learning in Predicting and Combating Antimicrobial Resistance. Comput Struct Biotechnol J (2025) 27:423–39. 10.1016/j.csbj.2025.01.006 39906157 PMC11791014

[249] Elalouf A Elalouf H Rosenfeld A Maoz H . Artificial Intelligence in Drug Resistance Management. 3 Biotech (2025) 15(5):126. 10.1007/s13205-025-04282-w 40235844 PMC11996750

[250] AlGain S Marra AR Kobayashi T Marra PS Celeghini PD Hsieh MK Can We Rely on Artificial Intelligence to Guide Antimicrobial Therapy? A Systematic Literature Review. Antimicrob Steward Healthc Epidemiol (2025) 5(1):e90. 10.1017/ash.2025.47 40226293 PMC11986881

[251] Kaushik R Kant R Christodoulides M . Artificial Intelligence in Accelerating Vaccine Development - Current and Future Perspectives. Front Bacteriol (2023) 2:1258159. 10.3389/fbrio.2023.1258159

[252] Masignani V Pizza M Moxon ER . The Development of a Vaccine Against Meningococcus B Using Reverse Vaccinology. Front Immunol (2019) 10:751. 10.3389/fimmu.2019.00751 31040844 PMC6477034

[253] Oxford Vaccine Research Group. Oxford Launches Major New AI Vaccine Research Programme with the Ellison Institute of Technology. Available online at: https://www.ovg.ox.ac.uk/news/oxford-launches-major-new-ai-vaccine-research-programme-with-the-ellison-institute-of-technology (Accessed September 08, 2025).

[254] Baddal B . Microfluidic Organ-Chips and Infectious Diseases: Insights from the Development and Applications Perspective. Cyprus J Med Sci (2022) 7(1):1–8. 10.4274/cjms.2020.1426

[255] Wang Y Gao Y Pan Y Zhou D Liu Y Yin Y Emerging Trends in Organ-on-a-Chip Systems for Drug Screening. Acta Pharm Sin B (2023) 13(6):2483–509. 10.1016/j.apsb.2023.02.006 37425038 PMC10326261

[256] Sunildutt N Parihar P Chethikkattuveli Salih AR Lee SH Choi KH . Revolutionizing Drug Development: Harnessing the Potential of Organ-On-Chip Technology for Disease Modeling and Drug Discovery. Front Pharmacol (2023) 14:1139229. 10.3389/fphar.2023.1139229 37180709 PMC10166826

[257] Leung CM de Haan P Ronaldson-Bouchard K Kim G-A Ko J Rho HS A Guide to the Organ-On-a-Chip. Nat Rev Methods Primers (2022) 2:33. 10.1038/s43586-022-00118-6

[258] Maschmeyer I Lorenz AK Schimek K Hasenberg T Ramme AP Hübner J A Four-Organ-Chip for Interconnected Long-Term Co-Culture of Human Intestine, Liver, Skin and Kidney Equivalents. Lab Chip (2015) 15(12):2688–99. 10.1039/c5lc00392j 25996126

[259] Lee Y Kim MH Alves DR Kim S Lee LP Sung JH Gut-Kidney Axis on Chip for Studying Effects of Antibiotics on Risk of Hemolytic Uremic Syndrome by Shiga Toxin-Producing *Escherichia coli* . Toxins (Basel) (2021) 13(11):775. 10.3390/toxins13110775 34822559 PMC8622205

[260] Payasi A Yadav MK Chaudhary S Aggarwal A . Evaluating Nephrotoxicity Reduction in a Novel Polymyxin B Formulation: Insights from a 3D Kidney-on-a-Chip Model. Antimicrob Agents Chemother (2024) 68(10):e0021924. 10.1128/aac.00219-24 39225483 PMC11459911

[261] Wadman M . FDA No Longer Has to Require Animal Testing for New Drugs. Science (2023) 379(6628):127–8. 10.1126/science.adg6276 36634170

[262] Piergiovanni M Leite SB Corvi R Whelan M . Standardisation Needs for Organ on Chip Devices. Lab Chip (2021) 21(15):2857–68. 10.1039/d1lc00241d 34251386

[263] Srivastava SK Foo GW Aggarwal N Chang MW . Organ-On-Chip Technology: Opportunities and Challenges. Biotechnol Notes (2024) 5:8–12. 10.1016/j.biotno.2024.01.001 39416695 PMC11446384

[264] Piergiovanni M Jenet A Batista Leite S Cangar O Mian L Maurer P Organ on Chip: Building a Roadmap Towards Standardisation. Luxembourg: Publications Office of the European Union (2021). ISBN 978-92-76-43354-5, JRC126163. 10.2760/819439

[265] Anon. Organ-on-a-Chip Technologies Network (2025). Available online at: https://www.organonachip.org.uk/ (Accessed July 01, 2025).

[266] Cook SR Ball AG Mohammad A Pompano RR . A 3D-Printed Multi-Compartment Organ-On-Chip Platform with a Tubing-Free Pump Models Communication with the Lymph Node. Lab Chip (2025) 25(2):155–74. 10.1039/d4lc00489b 39661075 PMC11633827

[267] UK Health Security Agency. Case Study: VDEC Pre-Clinical Team Helps to Develop Organ-On-a-Chip to Protect Human Health. (2025). Available online at: https://www.gov.uk/government/case-studies/vdec-pre-clinical-team-helps-to-develop-organ-on-a-chip-to-protect-human-health (Accessed July 01, 2025).

[268] Wang Q Yang Y Chen Z Li B Niu Y Li X . Lymph Node-On-Chip Technology: Cutting-Edge Advances in Immune Microenvironment Simulation. Pharmaceutics (2024) 16(5):666. 10.3390/pharmaceutics16050666 38794327 PMC11124897

[269] Ingber DE . Human Organs-On-Chips for Disease Modelling, Drug Development and Personalized Medicine. Nat Rev Gene (2022) 23(8):467–91. 10.1038/s41576-022-00466-9 35338360 PMC8951665

[270] Ardila CM Zuluaga-Gómez M Vivares-Builes AM . Applications of Lab on a Chip in Antimicrobial Susceptibility of *Staphylococcus Aureus*: A Systematic Review. Medicina (Kaunas) (2023) 59(10):1719. 10.3390/medicina59101719 37893437 PMC10608121

[271] HM Government. Confronting Antimicrobial Resistance 2024 to 2029 (2024). Available online at: https://www.gov.uk/government/publications/uk-5-year-action-plan-for-antimicrobial-resistance-2024-to-2029 (Accessed January 15, 2026).

[272] EU Directorate-General for Health and Food Safety. Council Recommendation on Stepping up EU Actions to Combat Antimicrobial Resistance in a One Health approach (2023). Available online at: https://health.ec.europa.eu/publications/council-recommendation-stepping-eu-actions-combat-antimicrobial-resistance-one-health-approach_en (Accessed January 15, 2026).

